# Development of gut microbiota during the first 2 years of life

**DOI:** 10.1038/s41598-022-13009-3

**Published:** 2022-05-31

**Authors:** Mona-Lisa Wernroth, Sari Peura, Anna M. Hedman, Susanne Hetty, Silvia Vicenzi, Beatrice Kennedy, Katja Fall, Bodil Svennblad, Ellika Andolf, Göran Pershagen, Jenny Theorell-Haglöw, Diem Nguyen, Sergi Sayols-Baixeras, Koen F. Dekkers, Stefan Bertilsson, Catarina Almqvist, Johan Dicksved, Tove Fall

**Affiliations:** 1 Department of Medical Sciences, Molecular Epidemiology and Science for Life Laboratory, Uppsala University, EpiHubben, MTC-huset, 751 85 Uppsala, Sweden; 2 Uppsala Clinical Research Center, Uppsala University, Uppsala, Sweden; 3 Department of Forest Mycology and Plant Pathology, and Science for Life Laboratory, Swedish University of Agricultural Science, Uppsala, Sweden; 4 Department of Ecology and Genetics, Limnology and Science for Life Laboratory, Uppsala University, Uppsala, Sweden; 5 Swedish Nuclear Fuel and Waste Management Co., (SKB), Stockholm, Sweden; 6 Department of Medical Epidemiology and Biostatistics, Karolinska Institutet, Stockholm, Sweden; 7 Department of Medical Sciences, Clinical Diabetology and Metabolism, Uppsala University, Uppsala, Sweden; 8 School of Medicine, University of Tasmania, Hobart, Australia; 9 Division of Biological Sciences, University of California, San Diego, USA; 10 Clinical Epidemiology and Biostatistics, School of Medical Sciences, Örebro University, Örebro, Sweden; 11 Integrative Epidemiology, Institute of Environmental Medicine, Karolinska Institutet, Stockholm, Sweden; 12 Department of Surgical Sciences, Uppsala University, Uppsala, Sweden; 13 Department of Clinical Sciences, Division of Obstetrics and Gynaecology, Danderyd Hospital, Karolinska Institutet, Stockholm, Sweden; 14 Institute of Environmental Medicine, Karolinska Institutet, Stockholm, Sweden; 15 Department of Medical Sciences, Respiratory, Allergy and Sleep Research, Uppsala University, Uppsala, Sweden; 16 Department of Aquatic Sciences and Assessment, Swedish University of Agricultural Sciences, Uppsala, Sweden; 17 Pediatric Allergy and Pulmonology Unit at Astrid Lindgren Children’s Hospital, Karolinska University Hospital, Stockholm, Sweden; 18 Department of Animal Nutrition and Management, Swedish University of Agricultural Sciences, Uppsala, Sweden

**Keywords:** Microbiota, Risk factors

## Abstract

Although development of microbiota in childhood has been linked to chronic immune-related conditions, early childhood determinants of microbiota development have not been fully elucidated. We used 16S rRNA sequencing to analyse faecal and saliva samples from 83 children at four time-points during their first 2 years of life and from their mothers. Our findings confirm that gut microbiota in infants have low diversity and highlight that some properties are shared with the oral microbiota, although inter-individual differences are present. A considerable convergence in gut microbiota composition was noted across the first 2 years of life, towards a more diverse adult-like microbiota. Mode of delivery accounted for some of the inter-individual variation in early childhood, but with a pronounced attenuation over time. Our study extends previous research with further characterization of the major shift in gut microbiota composition during the first 2 years of life.

## Introduction

Microbial colonization of the gut is essential for natural maturation of the intestine and immune system in infants, and early life imbalances in the gut microbiota have been suggested to increase the risk of chronic immune-related conditions such as allergy^1^ and type 1 diabetes^2^. Like the gut, the oral cavity also harbours a diverse microbial community and the composition of the oral microbiota has been associated with dental health^3^ and cardiovascular disease^4^.

Microbial colonization of the gut starts at birth, or even in utero^5^ and maternal vaginal, gut and skin constitute important bacterial inocula. The gut microbiota undergoes most of its development very early in life and major changes in the composition of the gut microbiota have been observed until the child is 2 to 3 years of age^2,6,7^. However, an adult level gut microbial diversity might not be reached even at 5 years of age^7^. The diversity and colonization patterns have been proposed to be influenced by factors such as mode of delivery^2^, having siblings^2^, gestational age^2^, birth weight^8^, antibiotics use during the first years of life^2^, presence of furry pets in the household^2^ and diet^2^. Mode of delivery is recognised as a major determinant of microbiota composition, and differentially delivered infants have contrasting gut microbiota composition throughout the first years of life^2,9–12^. However, longitudinal studies following children from birth through their first 2 years of life investigating the role of mode of delivery are scarce^9,12^. Further, prenatal factors such as exposure to antibiotics, might be of importance and a recent review by Grech et al.^13^ highlighted the lack of longitudinal studies investigating the effect of maternal exposures on gut microbiota development in children. Thus, there is a need to better understand the role of mode of delivery and other factors of importance for shaping the gut microbiota composition in a growing child.

A study of the oral and gut microbiota in 3-day-old children suggested that more species and strains are shared between their own oral and gut microbiota in children than in their mothers^14^. The duration of these similarities is still unclear and warrants further investigation.

The aim of the present study was to investigate environmental exposures of relevance for gut microbiota development in children, following them longitudinally from birth until age 2, and to describe associations between gut and oral microbiota. The study was based on repeated faecal and saliva samplings in a cohort of 83 children and their mothers and leveraged the unique availability of high-quality national registers and questionnaire data to obtain information on maternal and birth characteristics.

## Materials and methods

### The Born into Life cohort

**Figure 1:**
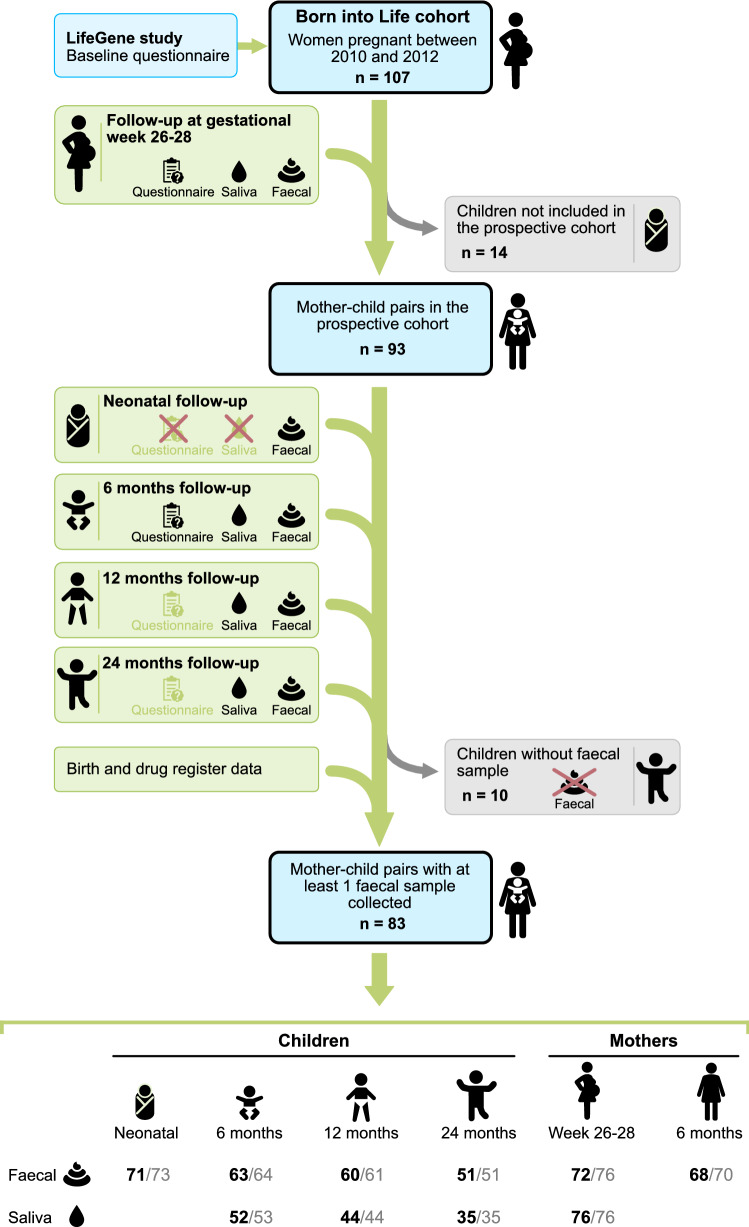
Flow chart of the study population. The cohort consists of pregnant women who lived in Stockholm, Sweden enrolled in the LifeGene study. After obtaining parental consent, the children were included in the prospective cohort (n = 93). A red cross indicates that questionnaires or saliva samples were not administrated. Light green questionnaire symbol indicates that the answers were not used in the current study. Number of samples with at least 12,588 reads reported in black font. Number of samples sequenced are reported in grey font. The figure is made by Andreas Dahlin at Visualize your Science.

This study is based on the prospective longitudinal cohort study Born into Life^15^. The cohort consists of women who lived in Stockholm County, Sweden, enrolled in the LifeGene study and became pregnant at any time between 2010 and 2012^16^. Children were delivered at the same clinic and were included in the cohort upon delivery. The cohort comprised 107 pregnant women, while the associated child cohort, with consent from the parents to follow the children also after birth, comprised 93 children (Fig. 1).

### Phenotypic information

Repeated questionnaires and linkage to national health registers were used to collect the phenotypic information. Linkage to national health registers was enabled through use of the unique individual Swedish personal identity number.

From the Medical Birth Register, we collected information relating to the birth (sex, date of birth, mode of delivery, gestational age, and birthweight) and the maternal characteristics such as parity, maternal body mass index (BMI), and smoking in early pregnancy. Information on exclusive breastfeeding was provided from the Born into Life questionnaire at 6 months. We obtained information on dispensed systemic antibiotics (ATC: J01) including date of dispense from the Swedish Prescribed Drug Register. Prenatal antibiotic exposure was defined as any maternal dispensed prescription of oral antibiotics during pregnancy. Information on exposure to furry pet, defined as presence of a cat or a dog in the household, was obtained from the questionnaire administrated at gestational week 26–28. We retrieved information on maternal highest education level from the LifeGene questionnaire.

### Faecal and saliva samples within the Born into Life cohort

Both faecal and saliva samples were collected following a standardized protocol^17^. Faecal samples were collected from the children 2–4 days after birth and at 6, 12, and 24 months of age, whereas maternal samples were collected at gestational week 26–28, and 6 months post-partum. The neonatal faecal samples were collected by the midwife at the routine health check-up after birth. The remaining faecal samples were collected by the parents the day before visits to the test centre. Faecal samples were kept frozen at home and transported to the test centre in a cool transport container to prevent thawing. Subsequently, faecal samples were stored at − 80 °C until processing. Saliva samples from children were collected in the evenings at 6, 12, and 24 months of age using cotton swabs placed into Salivette tubes (Salivette–Sarstedt), whereas samples from mothers were collected in Salivette tubes according to a standard protocol one evening during gestational week 26–28^18^. The saliva samples were stored overnight at standard refrigerator temperature. Ethical approval for the study was granted by the Regional Ethics Review Board in Stockholm, Sweden (reference numbers 2011/192–31/2, 2011/1127–31/3, and 2014/1081–32 with amendments) and complied with the World Medical Association Declaration of Helsinki regarding ethical conduct of research. Informed consent for each participant in the maternal cohort was obtained during enrolment into the study and both parents gave informed consent for their children.

### Microbiota analysis

The analysis order was randomly assigned across samples and DNA extraction was performed separately for faecal and saliva samples. DNA from 407 faecal samples (249 samples from children, 158 from mothers) and 241 saliva samples (156 samples from children, 85 samples from mothers) were all extracted using the MO BIO PowerSoil DNA kit (MO BIO, Solana beach, CA, USA) and the MiniG1600 homogeniser (SPEX SamplePrep, Metuchen, NJ, USA) according to the protocol provided by the manufacturer. Five negative controls were included in the saliva DNA extraction procedure. After PCR amplification of the v3-v4 regions of 16S rRNA gene (PCR protocol is available in^18^), the amplified fragments were purified with AMPure XP beads and DNA concentration was quantified using Qubit (ThermoFisher Scientific) with the dsDNA high-sensitivity assay. All samples and negative controls were subjected to gel electrophoresis to assess presence of gel bands and product size after each PCR amplification and purification step, and selected samples were also analysed using a bioanalyzer (Agilent Technologies, Santa Clara, CA, USA) to ensure high DNA quality and correct band size. Four of the negative controls showed no amplification, while the fifth showed some evidence of contamination. The final amplicon libraries were also verified by bioanalyzer analysis. In total, 407 faecal and 216 saliva samples with individual dual barcodes produced sufficient amount of product. The faecal samples were randomly allocated into three pools and saliva samples into two pools of 120–150 samples, each pooled in equimolar amounts. The pools were sequenced using Illumina MiSeq technology (v3 chemistry, 2*300 bp) at Science for Life Laboratory (Uppsala University, Sweden). A total of nine negative control samples were included in the sequencing, whereof eight had very low number of reads, and the previously suspected contaminated sample had a significant number of reads. The resulting sequence reads were processed using the software Mothur^19^ following the standard recommended procedure^20^, except that clustering of operational taxonomic units (OTUs) was done using VSEARCH (abundance-based greedy clustering)^21^ as implemented in Mothur. No batch effects with regards to sequencing pools were detected by principal coordinate analysis (PCoA). After visual inspection of the distribution of read counts, samples with fewer than 12,588 reads were removed (1 saliva sample and 9 faecal samples) and the remaining samples were rarefied to this sequencing depth using Mothur. A cut-off of 97% sequence identity was used to delineate OTUs. The taxonomic classification of OTUs was done using the SILVA database (version 132, released on Dec 13, 2017)^22–24^. Across more than 34 million pair-end reads, we observed 733,773 OTUs with 709,652 of these with less than 10 reads. These were discarded as recommended in Franzén et al.^25^ to reduce the number of spurious OTUs. The remaining 24,115 OTUs comprised 85% of the total amount of reads. The removed OTUs were most commonly classified as belonging to the family *Lachnospiraceae* (25%), unclassified *Clostridiales* (15%) and unclassified Bacteria (14%). Taxonomic classification based on SILVA was largely confirmed on genus level for the top 20 OTUs by BLAST to the NCBI 16S data base accessed on January 19, 2022.

We excluded 10 mother–child pairs because no faecal sample was collected for the child and our final study population therefore included 245 faecal samples and 131 saliva samples from 83 children, and 140 faecal samples and 76 saliva samples from 80 mothers (Fig. 1). In total 58 children had faecal samples from at least 3 sampling occasions and 38 from all 4 occasions.

### Statistical analysis

The gut microbiota was assessed with faecal samples and the oral microbiota with saliva samples.

#### Outcomes

We considered five different aspects of gut microbiota composition as outcomes: (1) alpha diversity (taxonomic diversity of microbiota within samples) assessed by the inverse Simpson index^26^, (2) beta diversity (overall community differences between samples) assessed by Bray–Curtis distance, (3) relative abundance of each bacterial phylum, (4) relative abundance of each bacterial family (only analysed if there was a significant association at the phylum level) and (5) relative abundance of the 20 most common OTUs among the children (Supplementary Table S1).

Alpha diversity and beta diversity were calculated at the OTU level with the R package *vegan*^27^. All analyses were performed in R version 4.0.0^28^. Beta diversity was illustrated by PCoA plots and these plots were for descriptive purposes only. The relative abundance of each bacterial phylum and family was calculated for those phyla and families present in at least 75% of the faecal samples. The most common OTUs were selected by rank-transforming OTU abundance within each sample (with absent OTUs given the lowest possible rank), and choosing the 20 OTUs with the highest average rank across all samples. These 20 OTUs represented 51% of the reads in the faecal samples from children.

#### Distance-based similarity analysis

To understand the microbial communities shared between mothers and their children, as well as between unrelated individuals (non-self comparisons), and between faecal and saliva samples, we calculated Bray–Curtis and Jaccard distances between the samples. For non-self comparisons, we first calculated the distance for all possible relevant comparisons, and thereafter calculated the mean distance per individual. Lastly, we calculated the mean value with 95% confidence interval (CI) and performed t-tests for all possible comparisons. Results with a two-sided p < 0.05 were considered to be statistically significant and no adjustments for multiple comparisons were performed.

#### Exposures

We investigated the following potential determinants of child gut microbiota composition as exposures: age, maternal gut microbiota composition at gestational week 26–28, mode of delivery (vaginally and by caesarean section), parity, gestational age, birth weight, antibiotic treatment (both prenatal and postnatal), and the presence of furry pet in the household.

Potential confounders of each association were selected for adjustment using the d-separation criteria applied on Directed Acyclic Graphs, DAGs^29,30^, taking into account prior knowledge regarding their effect on the exposure and gut microbiota (Supplementary Fig. S1). We identified the potential confounders for each model separately.

Factors associating with the oral microbiota in this cohort have been previously analysed in^18^ and will thus not be presented here.

#### Gut microbiota—models

The outcomes alpha diversity and relative abundances were analysed using ordered logistic regression models (cumulative logit)^31^. To take the repeated measurements design into account, clustered robust standard errors with participant identity as cluster were used. The outcome beta diversity was analysed using the permutational multivariate ANOVA (PERMANOVA) method^32^, as implemented in the R package *vegan*. To calculate the type II sum of squares, the wrapper function *adonis_II* from the R package *RVaideMemoire*^33^ was used. The PERMANOVA analyses were performed for each sampling time-point separately. The homogeneity of within-group variation was checked using PERMDISP.

The gut microbiota development over time in children was analysed using statistical models with age of the child as the only independent variable. Age was modelled using restricted cubic splines with 4 knots at the 5th, 35th, 65th, and 95th percentiles to allow for nonlinear associations.

To investigate the effect of the exposures on gut microbiota development in children, we fitted models including the exposure, age, the interaction between exposure and age, and potential confounders (Table 1).

**Table 1:** Outcomes, exposure, and confounders included in the statistical models.

Outcome	Exposure	Confounders
Child gut microbiota at birth, 6 m, 12 m and 24 m	Age	
	Maternal gut microbiota at gestational week 26–28	Furry pet, parity
	Mode of delivery	Maternal BMI, parity, gestational age, birth weight
	Parity	Maternal age
	Gestational age	Maternal education level*
	Birth weight	Maternal BMI, maternal education level, parity, gestational age*
Child gut microbiota at birth, 6 m, 12 m and 24 m	Prenatal antibiotic treatment (maternal use of antibiotics during pregnancy)	Maternal age, maternal education, parity, maternal BMI*
Child gut microbiota at 24 m	Antibiotic treatment	Maternal age, maternal education level, sex, gestational age, mode of delivery, parity, birth weight
Child gut microbiota at birth, 6 m, 12 m and 24 m	Furry pet in the household at baseline	Maternal age, maternal education level

*BMI* Body Mass Index, *m* month.

*Maternal smoking during pregnancy was identified as a potential confounder. However, in our maternal cohort no women reported smoking whilst pregnant, and this variable is therefore not included in the model.

Continuous exposures were included as restricted cubic splines, with 3 knots at the 10th, 50th, and 90th percentiles. The interaction was based only on the linear part of the spline for age of the child, gestational age, birth weight, and maternal gut microbiota composition. When the p for the interaction was ≥ 0.05 (no multiple testing correction) the model was refitted without the interaction. Ordinal regression model-based predictions of mean relative abundance of phyla, families and OTUs were calculated and plotted for models including a continuous exposure and/or an interaction, otherwise only the odds ratio (OR) was reported.

The number of children exposed to antibiotics during the first year of life was low (n = 7) in our study. We therefore restricted our analysis on any association between antibiotics and the gut microbiota composition sampled at 24 months.

To evaluate the maternal gut microbiota composition as an exposure, alpha diversity, relative abundance of phyla, families and relative abundance of the 20 most common OTUs in gut microbiota in children were compared in turn as outcomes (e.g. maternal alpha diversity was explored in association with child alpha diversity).

The results from the gut microbiota models (Table 1) including four different aspects of gut microbiota composition as outcomes [(1) alpha diversity, (2) beta diversity (3) phylum, and (4) OTU], were then evaluated jointly for statistical significance using a false discovery rate (FDR) of 10%, where FDR was calculated using the Benjamini–Hochberg method^34^. The FDR cut-off corresponded to a nominal p of 0.0213. Findings driven by a single influential observation were not considered or discussed further but are reported in Supplementary Table S2. Family level analyses were only performed if there was a significant association at the phylum level.

## Results

Of the 83 children (50 boys and 33 girls), 15 children were delivered by caesarean section (10 emergency, 4 elective, 1 missing information on type) (Table 2). The median birth weight was 3,530 g, and the median gestational age was 40 weeks. Overall, 18 children were exposed to prenatal antibiotic treatment and among them 8 children were exposed ≤ 90 days before birth. At the 12 months sampling occasion, 7 children had been prescribed antibiotics, and 15 children at the 24 months sampling occasion. In the latter group 6 children were exposed within 90 days before the sampling. Children delivered by caesarean section were not overrepresented in the group of children treated with antibiotics, only 1 child delivered by caesarean section had antibiotic treatment prescribed before the 12 months sampling occasion. Overall, we detected 17,757 and 5,326 OTUs (OTUs with at least 10 reads) in faecal and saliva samples of the children, respectively.

**Table 2:** Descriptive characteristics of the 83 mother–child pairs included in the study.

	Mother–child pairs n = 83	Missing data^a^ n
**Maternal characteristics**		
Age at childbirth (years), median (Q1, Q3)	32.3 (30, 35)	0
University level education, n (%)	71 (86)	6
BMI in the first trimester (kg/m^2^), n (%):		0
Underweight (< 18.5)	2 (2)	
Normal weight (18.5–24.9)	62 (75)	
Overweight (25–29.9)	16 (19)	
Obese (≥ 30)	3 (4)	
Smoking during pregnancy, n (%)	0 (0)	2
Multipara, n (%)	26 (31)	0
Dispensed antibiotic prescription during pregnancy, n (%):	18 (22)	
**Birth characteristics**		
Caesarean delivery, n (%)	15 (18)	0
Gestational age (weeks), n (%):		0
Preterm (< 37)	5 (6)	
Full term (37–41)	73 (88)	
Post term (≥ 42)	5 (6)	
Birth weight (grams), median (Q1,Q3)	3,530 (3,230, 3,810)	0
**Child characteristics**		
Male, n (%)	50 (60)	0
Age at follow-up (months), median (Q1,Q3)		0
Neonatal	0.10 (0.07, 0.20)	
6 months	6.5 (6.1, 6.9)	
12 months	12.1 (11.8, 12.8)	
24 months	24.2 (24.0, 25.0)	
Age when exclusive breastfeeding was discontinued, n (%):^b^		32
0–2 months	4 (8)	
3–4 months	14 (27)	
≥ 5 months	33 (65)	
Dispensed antibiotic prescription before age 2, n (%):	15 (18)	0
**Other characteristics**		
Furry pet present in household, n (%)	12 (21)	25

Q1: First Quartile. Q3: Third Quartile.

*BMI* Body Mass Index.

^a^Information on smoking during pregnancy was not recorded in the Medical Birth Register. Mothers did not answer the questions on education, breastfeeding or presence of furry pet.

^b^We did not perform an analysis of a potential association between breastfeeding and gut microbiota as children who were exclusively breast-fed for ≥ 5 months may have been introduced to foods at the 6 months sampling occasion. Breastfeeding also had a high number of missing values.

### Gut microbiota became more homogeneous with age of the child

We observed an increase in alpha diversity of the gut microbiota with increasing age of the children (p < 0.001, Supplementary Fig. S2, Supplementary Table S2).

**Figure 2:**
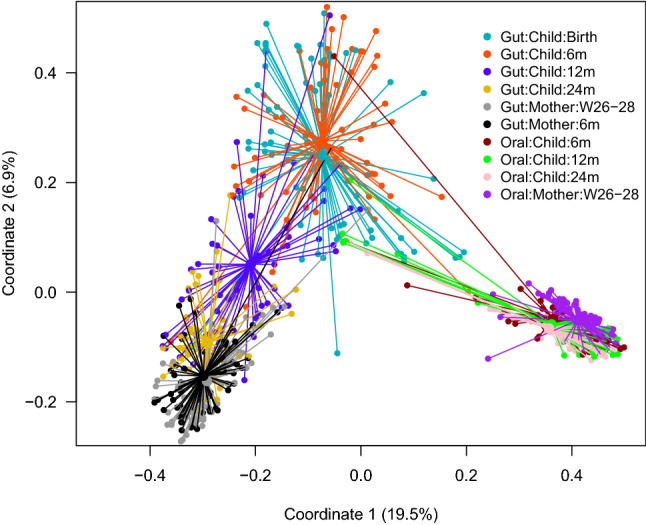
Principal coordinate analysis plot (Bray–Curtis distances) illustrating the children and their mother’s overall composition of the gut and oral microbiota at different time points, from birth (neonatal sample) of the child to 24 months and from mothers at 26–28 weeks of gestation and 6 months after delivery. *W* gestational week and *m* month.

**Figure 3:**
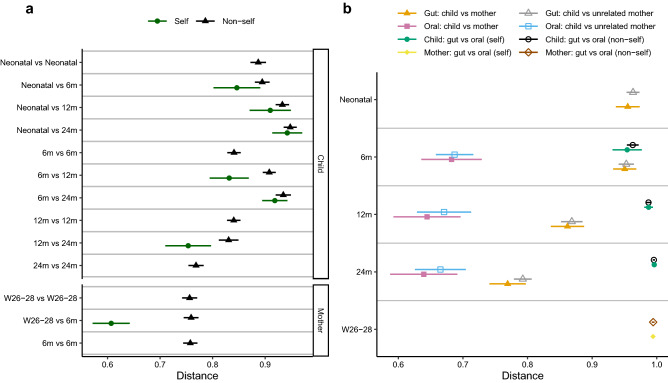
Comparison of microbiota composition based on Bray–Curtis distances. The shorter the distance between any pair of comparisons indicate greater similarity between microbial communities collected at those times. *W* gestational week and *m* month. Symbols represent means and error bars 95% confidence intervals. (**a**) Comparisons of gut microbiota at different sampling times, where distances between different sampling points within the same child/mother (self), and distances between unrelated children (non-self) and between unrelated mothers (non-self) are denoted. (**b**) Comparisons between gut and oral microbiota, and between children and their mothers and unrelated mothers. The distance between child and mother is based on the maternal sample at gestational week 26–28.

Figure 2 illustrates the children and their mother’s overall composition of the gut microbiota at different time points. The Bray–Curtis distance-based similarity analysis indicated that the largest changes in microbiota composition occurred during the first year of life (Fig. 3a, Supplementary Table S3). For example, the self-comparison between neonatal and 12 months showed a larger difference in composition than the comparison between 12 and 24 months (p < 0.001, Fig. 3a, Supplementary Table S3 and S5), despite the same 12-month timespan between samplings. However, all of the self-comparisons across time in children were found to be less similar than self-comparison in mothers (p < 0.001, Fig. 3a, Supplementary Table S3 and S5), indicating a more stable microbiota in mothers, despite the pregnancy. Furthermore, the non-self comparison presented in Fig. 3a indicated that the Bray–Curtis distance between faecal samples from different children also became smaller with age, and the similarity at 24 months was for example higher than at 12 months (p < 0.001, Fig. 3a, Supplementary Table S3 and S5). The similarity to the mothers’ samples also increased with age (Fig. 3b, Supplementary Table S4) and the similarity at 24 months was for example higher than the similarity at 12 months (p < 0.001, Supplementary Table S6) In summary, these results indicate that the gut microbiota in children matured with age and became less variable between different children as compared to the earlier stages of their lives.

The results based on the Jaccard distance are presented in Supplementary Fig. S3, Table S3, S4, S7 and S8. The mean Jaccard distance was in general lager than the Bray–Curtis distance, but the same pattern for comparisons between groups was observed independent of the metric.

**Figure 4:**
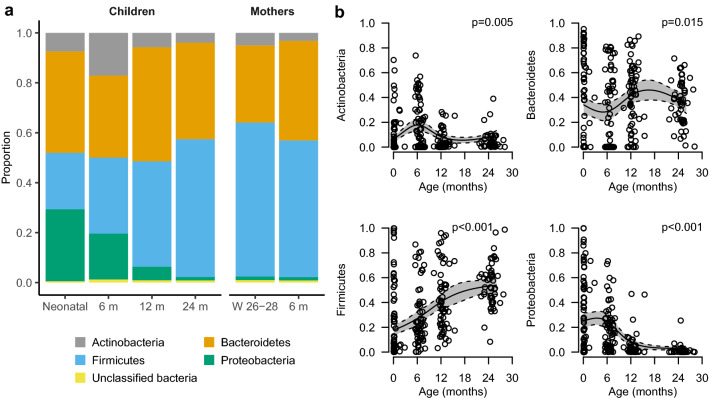
Bacterial phyla present in at least 75% of the faecal samples. **a** Stacked bar charts of the relative distribution of phyla detected in the gut at different time points. (**b**) Model-based predictions of relative abundance of phyla in the gut of children plotted against age. Lines indicate mean values (solid) and 95% confidence intervals (dashed). The y-axis shows the relative abundance in proportion. The predictions are shown from the 10th to the 90th percentile of age. *W* gestational week and *m* month.

At the phylum level we noted an association of age with an increase in relative abundance of Firmicutes (p < 0.001), a decrease of Proteobacteria (p < 0.001), an inverse U-shaped association with highest prevalence at 6 months for Actinobacteria (p = 0.005) and a non-linear association between Bacteroidetes and age (p = 0.015) (Fig. 4a,b). At 24 months, the phylum distribution in children was similar to the mothers. The analysis at family level indicated that the observed association with age was mainly driven by *Bifidobacteriaceae* for the phylum Actinobacteria and by *Enterobacteriaceae* for the phylum Proteobacteria (Supplementary Fig. S4). For Firmicutes no single major contributor at the family level was observed (Supplementary Fig. S4).

Age was associated with 18 of the 20 most common OTUs (Supplementary Fig. S5 and S6, Supplementary Table S2). *Faecalibacterium* (OTU8), *Blautia* (OTU22), *Agathobacter* (OTU13), and *Anaerostipes* (OTU31) were initially rare, but appeared at 12 and 24 months; these OTUs were present also in mothers (Supplementary Fig. S5).

### Similarity between gut and oral microbiota

All of the 20 most abundant OTUs detected in faecal samples from the children were also present in at least one of the saliva samples from children. *Streptococcus* (OTU3, OTU17), *Veillonella* (OTU5) and *Haemophilus* (OTU6) were present in at least 93% of the saliva samples, while the other 16 OTUs were present in 2% to 34% of the samples. The median number of detected OTUs in samples from children (age 6 to 24 months) was 590 and 488 in faecal and saliva samples, respectively.

We compared the gut microbiota composition and oral microbiota composition at 6, 12 and 24 months and the distance-based similarity analysis showed a small increase in Bray–Curtis distance with age (24 months vs 6 months p < 0.001 and 24 months vs 12 months p = 0.013, Fig. 3b, Supplementary Table S4 and S6). However, already at 6 months of age, the mean Bray–Curtis distance was as large as 0.95 (95% CI: 0.93–0.98) increasing to 1.00 (0.99–1.00) at 24 months (Supplementary Table S4). The same pattern was observed for the Jaccard distance (Supplementary Table S4 and S8). The median number of shared OTUs between faecal and saliva samples was 13.5, 9.0, and 8.0 at 6, 12, and 24 months in children, respectively, and 6.0 shared OTUs in mothers. *Veillonella* (OTU5) was the most frequently shared OTU between faecal and saliva samples at 6 months of age. It was detected in both the faecal and saliva sample in 92% (46/50), 82% (36/44) and 43% (15/35) of the children at 6, 12, and 24 months (Supplementary Table S9), respectively, and in 39% (26/67) of the mothers (Supplementary Table S10). The median abundance of shared OTUs in faecal samples decreased from 9.5% at 6 months to 2.6% at 24 months and was then similar in abundance in mothers (median: 2.2%). This indicates that the gut and oral microbiota share some characteristics in infants, but that with time these communities become more distinct. We observed no differences between self-comparisons and non-self comparisons, of oral and gut microbiota, at any of the sampling occasions (Children: 6 months p = 0.97, 12 months p = 0.95, 24 months p = 0.62; Mothers p = 0.91, Fig. 3b, Supplementary Tables S4 and S6).

### Mode of birth differentiated gut microbiota in children

**Figure 5:**
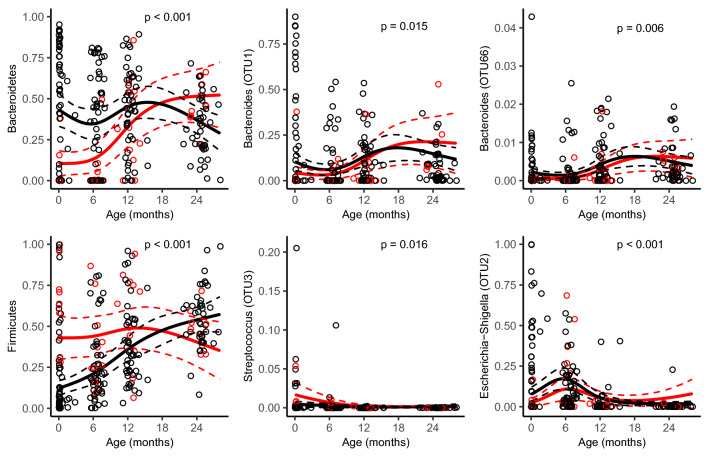
Model-based predictions of relative abundance in bacterial phyla and specific operational taxonomic units (OTUs) in the gut microbiota by mode of delivery plotted against age. Lines indicate mean values (solid) and 95% confidence intervals (dashed), adjusted for potential confounders (see Table 1). P reflect for the interaction between mode of delivery and child age. All continuous adjustment variables are fixed at their median value and all categorical adjustment variables are fixed at their most common category.

The composition of gut microbiota of a child was almost as similar to the microbiota of an unrelated mother as to their own mother (Fig. 3b). We observed an inverse association between the mother’s and child’s relative abundance of the phylum Bacteroidetes (Supplementary Fig. S7, Supplementary Table S2) and a positive association for an OTU belonging to the genus *Veillonella* (OTU5) (Supplementary Fig. S8, Supplementary Table S2). The overall gut microbiota composition differed between children delivered vaginally and by caesarean section at birth (PERMANOVA p = 0.007, PERMDISP = 0.46, Supplementary Fig. S9), but not at later sampling occasions. Compared with vaginally delivered children, children delivered by caesarean section had a lower relative abundance of Bacteroidetes and the strength of the associations decreased with age (Fig. 5). The same type of age-dependent association was also observed for two OTUs belonging to the *Bacteroides* genus (OTU1 and OTU66) (Fig. 5). Children delivered by caesarean section also had a lower abundance of an additional OTU belonging to the *Bacteroides* genus (OTU29 (OR = 0.32, p = 0.009)), but no significant interaction between mode of delivery and age was observed. The family level analysis suggests *Bacteroidaceae* to be a major contributor of the difference observed for Bacteroidetes (Supplementary Fig. S10). Delivery by caesarean section was positively associated with the relative abundance of Firmicutes (Fig. 5), *Veillonella* (OTU5, OR = 2.19, p = 0.009), *Faecalibacterium* (OTU8, OR = 2.15, p = 0.003) and *Streptococcus* (OTU3 (Fig. 5) and OTU17 (OR = 2.28, p = 0.016)). However, as *Veillonella* (OTU5) and *Faecalibacterium* (OTU8) represented a minor fraction of the abundance in neonatal samples, it is likely that other OTUs within the Firmicutes phylum are the main contributors to the differences observed in the Firmicutes. Either could any family be identified a main contributor even though we observed an association with *Streptococcaceae* (Supplementary Fig. S10). Furthermore, delivery by caesarean section was negatively associated with *Escherichia*-*Shigella* (OTU2) (Fig. 5).

### Diminished association of some factors with gut microbiota with child age

First-born children had a higher relative abundance of *Haemophilus* (OTU6, OR = 2.11, p = 0.005) than later-born children. Gestational age was negatively associated with Firmicutes (age p_interaction_ = 0.016) and *Streptococcus* (OTU3, age p_interaction_ = 0.021) and the strength of the associations decreased with age (Supplementary Fig. S11). Birth weight was associated with the overall gut microbiota composition of the child at the age 1 year (PERMANOVA p = 0.020) and two OTUs belonging to the genus *Bacteroides* (OTU1: p = 0.007, OTU29: p = 0.013, Supplementary Fig. S12).

Prenatal exposure to antibiotics was associated with a lower abundance of *Blautia* (OTU22, OR = 0.47, p = 0.016). We did not find any association between antibiotic treatment during the first 2 years of life and gut microbiota of the child at the age 2 years.

The presence of a furry pet in the household was associated with a lower relative abundance of the phylum Actinobacteria and an OTU belonging to the genus *Bifidobacterium* (OTU21) in the child (Supplementary Fig. S13). However, these associations diminished with age as there was an interaction between pet exposure and age of the child (Actinobacteria: p_interaction_ = 0.012, OTU21: p_interaction_ < 0.001). Furthermore, the family level analysis suggests *Bifidobacteriaceae* as the major contributor of the difference observed for Actinobacteria (Supplementary Fig. S14) between pet exposed and non-exposed children.

## Discussion

In this Swedish cohort of 83 children, we observed an age-dependent development of the gut microbiota, with an adult-like microbial composition attained around the age of 2 years. Our distance-based similarity analyses indicated that the similarity between the gut and oral microbiota decreased after 6 months of age, although already at this age, the microbiota were highly dissimilar, and the two communities were clearly different at 2 years. Mode of delivery differentiated the overall gut microbiota composition. Caesarean section was associated with a lower relative abundance of the phylum Bacteriodetes, with the family *Bacteroidaceae* being the major contributor and a higher relative abundance of the phylum Firmicutes. As expected, these associations were strongest during the first 6 months of life and diminished thereafter.

A major part of the maturation of the microbiota composition seems to occur in children at an early age. However, a recent study showed that several bacterial taxa are acquired late in childhood and that differences in microbiome structure and diversity still may exist even at 5 years of age^7^. In concordance with previous studies, we observed a gradual shift in the gut microbiota composition from birth to age 2, with an increased alpha diversity, increased relative abundance of the phylum Firmicutes and decreased relative abundance of the phylum Proteobacteria and Actinobacteria^2,9,12^. We found the most marked changes to occur during the first year of life. Even though the alpha diversity increased with age, the beta diversity was reduced and more similar to a characteristically adult community composition. Our results also suggest that the gut microbiota composition is rather stable in mothers compared to children, even when comparing samples during and after pregnancy. This finding aligns with a longitudinal study (n = 128 mother–infant pairs) that compared faecal samples from the same individual across time and found samples from mothers 2 months before and after delivery to be more similar than samples from children (2 months vs 6 months)^35^.

A study, by Ferretti et al.^14^, of 25 mother–child pairs reported that the number of shared oral—gut species decreased from an average of 9.8 at day 1 to 7.2 species already at 3 days of life, which can be compared with 6 shared oral—gut species in mothers. In addition, faecal samples were reported to have a lower abundance of oral-gut microbiota shared species in mothers than in infants. Our distance-based similarity analyses extends this finding and indicates that the similarity between oral and gut microbiota continue to decrease also after 6 months of age. Ferretti et al.suggested that oral bacterial transition to gut is more relevant in infants than in adults and speculated that this could be a consequence of reduced acid production in children at birth^14^. However, this could also be due to niche adaptations of the bacterial communities both in the oral cavity and the gut, as these communities develop early in life^6,36^. One example is *Bifidobacterium dentum* which have shown adaptation to the intestine via its acid tolerance, mucus adhering properties and metabolic plasticity towards dietary nutrient sources commonly found in the intestine^37^. In the present study, we lack saliva samples from children before 6 months of age and future work focusing on this period would likely advance the understanding of mechanisms of early microbiota acquisition and development. This could also enable identification of specific environmental sources of microbial inoculation.

Gut microbiota composition appears to be shaped by different factors and is modulated by age over time. In line with previous work, our study identified an association between caesarean section delivery and the overall gut microbiota composition^7,9,10^. However, no association with alpha diversity was observed. The negative association of delivery by caesarean section with abundance of *Bacteroides* has been previously observed^2,9,12^, but while this persisted throughout the first 6 months of life in the present study, the effects then diminished with age. *B. dentium* species are important regulators of the intestinal immunity^38,39^ and low abundances have been proposed to partially explain the associations between birth by caesarean section and adverse health effects such as asthma, allergies and atopy^40^. Our results also add to earlier findings showing that delivery by caesarean section is positively associated with bacteria affiliated with the phylum Firmicutes^9^, including the genera *Veillonella*^9,11^ and *Streptococcus*^11^. However, two large longitudinal studies^2,41^, did not show an association with either of these genera*. Veillonella* is commonly present in the gastrointestinal tract, mouth, vagina, and breast milk acting as an important link to the immune system^42^. While the overall patterns suggest that caesarean section has some effect on the development of the gut microbiota composition, presence of different species may be differentially affected.

Mother-to-child transmission of bacteria has been thought to be an important source of bacterial inoculum in children, and earlier studies have reported such transmission of bacterial strains^11,43^. We did not however observe strong evidence for higher bacterial community similarity within related mother–child pairs compared to unrelated pairs, neither in the gut nor in the oral microbial communities. Other studies performing community similarity analysis based on OTU level have reported comparable results^9,44–47^. However, to accurately quantify microbial transmission, strain level profiling is necessary, and we might have underestimated the difference between related and unrelated pairs as individuals frequently share common OTUs. Moreover, it should be noted that the confidence intervals were wide and the power low for detecting moderate similarities.

It is plausible that the impact of mode of delivery on infant gut microbiota development may be at least in part due to the lack of contact with maternal vaginal and intestinal microbiota for children delivered by caesarean section. In addition, differential occurrence of foetal exposure to maternal peripartal antibiotics prophylaxis, with potential effects on postpartum infant microbial colonization, could also mediate the association. Peripartal antibiotics prophylaxis was recommended for all mothers undergoing emergency caesarean section (n = 10 mothers in our study), whilst only on medical indication in elective caesarean section (n = 4) and vaginal deliveries (n = 68). We cannot ascertain use or timing of peripartal antibiotic prophylaxis in our maternal cohort, but a randomized control trial including 20 children in each arm with up to 3 years of follow-up failed to show any effect of different timing of antibiotic treatment, although both groups differed compared with a third group of vaginally delivered children. These findings indicate that there are other more important factors that explain the association of caesarean section with the child’s gut microbiota^48^.

Discrepancies between studies might be explained by different sampling schemes, sample sizes, hospital routines and methodologically also to the specific diagnostic regions of 16S rRNA gene that are being used. In addition, we performed the analysis of relative abundance at OTU level, while many of the other studies only compared responses at the broader genus level^2,11,12,41^.

We noted that prenatal exposure to antibiotics was associated with a lower relative abundance of an OTU belonging to the genus *Blautia*, which has been associated with potential probiotic functions^49^. These findings are supported by a cohort study on intrapartum antibiotics, including faecal samples collected at 6 weeks and 1 year of age from 266 children^50^. One explanation for the observed associations, in the present study, with prenatal antibiotics exposure could be changes in the gut and/or vaginal microbiota in the mothers that affect the child at birth or later^46^. Furthermore, the microbial colonization process could be started already in utero^5^, a process that may be affected by antibiotics administered during pregnancy.

In line with a large longitudinal study^2^, we observed that having a furry pet in the household was associated with a lower relative abundance of an OTU within the genus *Bifidobacterium*. However, our result should be interpreted with caution as information on furry pet was missing for 25 children which could bias the result.

The primary strengths of our study are the combination of oral and gut microbiota, its longitudinal design, following children from birth until age 2, and the use of data from high-quality registers with objectively and prospectively recorded information about maternal and birth characteristics. Some limitations apply. Our platform for 16S rRNA amplicon sequencing did not allow identification of the gut and oral bacteria to the species level and we were not able to characterize the functional properties of microbial changes associated with different exposures. Moreover, we did not investigate the effect of diet on gut microbiota or its possible role as a mediator as the dietary questionnaire information had several limitations. These limitations were (1) 32 mothers did not answer the question at age 6 months regarding diet (2) the pre-determined wide age-categories in the questionnaire (3) only four children in our study were exclusively breastfed for < 3 months, (4) no information of whether breast milk was substituted with solid foods or formula was available and (5) children who were exclusively breast-fed for ≥ 5 months may have been introduced to foods at the 6 months sampling occasion. The inability to include this information in the analysis prevented assessment as to whether diet could mediate the association of mode of delivery with bacterial composition. Although we adjusted the analyses for maternal and birth characteristics, residual confounding may still be present as we did not account for lifestyle factors and our sample size did not allow us to investigate any interactions between exposures. Furthermore, our study only included five children born pre-term (< 37 weeks) and one child with low birth weight (< 2500 g), limiting our power to investigate associations between gestational age and birth weight with gut microbiota variation in children. Additionally, previously decided sampling times did not take the time of antibiotic exposure into account and the low number of children exposed to antibiotics after birth limited our power to detect an association between antibiotics and gut microbiota variation in children. Furthermore, we could not compare different caesarean section types as only four children were delivered by elective caesarean section in our study. For the saliva samples, the overnight refrigerator storage of the samples may have caused some changes in composition. An earlier pilot study showed that room temperature storage of salivary samples for 6 h did not cause any major change in composition, while they noted some changes in specific bacterial taxa levels^51^. Such changes may have affected our ability to detect similarities between samples in terms of OTU levels. Finally, our study population represents a well-educated population from a predominantly urban area. This may restrict the generalizability of our findings as earlier studies have indicated that differences in geographical^6^ and socio-economic status^52^ might influence gut microbiota.

The results from our study, including both repeated saliva and faecal samples from children up to age 2 and from their mothers suggest that (1) the gut microbiota in infants are low in diversity with differences across individuals with regards to composition. (2) During the first 2 years of life, there is notable convergence in community composition towards a more diverse adult-like composition. (3) Oral and gut microbiota communities are distinct in composition in 2-year-old children, but share some similarities at age 6 and 12 months. (4) In infants, perinatal factors, such as mode of delivery, account for some of the observed differences between individuals but the association is attenuated with age.

## Supplementary Information

Supplementary Information.

## Data Availability

Restrictions apply to the availability of individual level health data, which were used under license and ethical approval for the current study and are not publicly available. Individual level data are however available from the authors upon reasonable request and with permission of the Swedish Ethical Review Authority. Bacterial sequencing data from saliva samples is available at the Sequence Read Archive (SRA) open repository under reference number PRJNA575550.

## References

[1] Bisgaard H (2011). Reduced diversity of the intestinal microbiota during infancy is associated with increased risk of allergic disease at school age. J. Allergy Clin. Immunol..

[2] Stewart CJ (2018). Temporal development of the gut microbiome in early childhood from the teddy study. Nature.

[3] Wang Y (2019). Oral microbiome alterations associated with early childhood caries highlight the importance of carbohydrate metabolic activities. mSystems.

[4] Slocum C, Kramer C, Genco CA (2016). Immune dysregulation mediated by the oral microbiome: Potential link to chronic inflammation and atherosclerosis. J. Intern. Med..

[5] Rackaityte E (2020). Viable bacterial colonization is highly limited in the human intestine in utero. Nat. Med..

[6] Yatsunenko T (2012). Human gut microbiome viewed across age and geography. Nature.

[7] Roswall J (2021). Developmental trajectory of the healthy human gut microbiota during the first 5 years of life. Cell Host Microbe.

[8] Chi C (2019). Longitudinal gut bacterial colonization and its influencing factors of low birth weight infants during the first 3 months of life. Front. Microbiol..

[9] Bokulich NA (2016). Antibiotics, birth mode, and diet shape microbiome maturation during early life. Sci. Transl. Med..

[10] Reyman M (2019). Impact of delivery mode-associated gut microbiota dynamics on health in the first year of life. Nat. Commun..

[11] Backhed F (2015). Dynamics and stabilization of the human gut microbiome during the first year of life. Cell Host Microbe.

[12] Jakobsson HE (2014). Decreased gut microbiota diversity, delayed bacteroidetes colonisation and reduced th1 responses in infants delivered by caesarean section. Gut.

[13] Grech A (2021). Maternal exposures and the infant gut microbiome: A systematic review with meta-analysis. Gut Microbes.

[14] Ferretti P (2018). Mother-to-infant microbial transmission from different body sites shapes the developing infant gut microbiome. Cell Host Microbe.

[15] Smew AI (2018). Limited association between markers of stress during pregnancy and fetal growth in 'born into life', a new prospective birth cohort. Acta Paediatr..

[16] Almqvist C (2011). Lifegene: A large prospective population-based study of global relevance. Eur. J. Epidemiol..

[17] Peura S (2018). Normal values for calprotectin in stool samples of infants from the population-based longitudinal born into life study. Scand. J. Clin. Lab. Invest..

[18] Kennedy B (2019). Oral microbiota development in early childhood. Sci. Rep..

[19] Schloss PD (2009). Introducing mothur: Open-source, platform-independent, community-supported software for describing and comparing microbial communities. Appl. Environ. Microbiol..

[20] Kozich JJ, Westcott SL, Baxter NT, Highlander SK, Schloss PD (2013). Development of a dual-index sequencing strategy and curation pipeline for analyzing amplicon sequence data on the miseq illumina sequencing platform. Appl. Environ. Microbiol..

[21] Rognes T, Flouri T, Nichols B, Quince C, Mahe F (2016). Vsearch: A versatile open source tool for metagenomics. PeerJ.

[22] Glockner FO (2017). 25 years of serving the community with ribosomal rna gene reference databases and tools. J. Biotechnol..

[23] Quast C (2013). The silva ribosomal rna gene database project: Improved data processing and web-based tools. Nucleic Acids Res..

[24] Yilmaz P (2014). The silva and "all-species living tree project (ltp)" taxonomic frameworks. Nucleic Acids Res..

[25] Franzén O (2015). Improved otu-picking using long-read 16s rrna gene amplicon sequencing and generic hierarchical clustering. Microbiome.

[26] Simpson E (1949). Measurement of diversity. Nature.

[27] Oksanen, J. *et al.**Vegan: Community Ecology Package* (2019).

[28] R Core Team (2020). R: A Language and Environment for Statistical Computing.

[29] Greenland S, Pearl J, Robins JM (1999). Causal diagrams for epidemiologic research. Epidemiology.

[30] Textor J, van der Zander B, Gilthorpe MS, Liskiewicz M, Ellison GT (2016). Robust causal inference using directed acyclic graphs: The r package 'dagitty'. Int. J. Epidemiol..

[31] Liu Q, Shepherd BE, Li C, Harrell FE (2017). Modeling continuous response variables using ordinal regression. Stat. Med..

[32] Anderson MJ (2001). A new method for non-parametric multivariate analysis of variance. Austral. Ecol..

[33] Hervé, M. *Rvaidememoire: Testing and Plotting Procedures for Biostatistics* (2020).

[34] Benjamini Y, Hochberg Y (1995). Controlling the false discovery rate: A practical and powerful approach to multiple testing. J. R. Stat. Soc. B.

[35] Hesla HM (2014). Impact of lifestyle on the gut microbiota of healthy infants and their mothers-the aladdin birth cohort. FEMS Microbiol. Ecol..

[36] Lif Holgerson P, Ohman C, Ronnlund A, Johansson I (2015). Maturation of oral microbiota in children with or without dental caries. PLoS ONE.

[37] Engevik MA (2021). The metabolic profile of bifidobacterium dentium reflects its status as a human gut commensal. BMC Microbiol.

[38] Kelly D (2004). Commensal anaerobic gut bacteria attenuate inflammation by regulating nuclear-cytoplasmic shuttling of ppar-gamma and rela. Nat. Immunol..

[39] An D (2014). Sphingolipids from a symbiotic microbe regulate homeostasis of host intestinal natural killer t cells. Cell.

[40] Sandall J (2018). Short-term and long-term effects of caesarean section on the health of women and children. Lancet.

[41] Galazzo G (2020). Development of the microbiota and associations with birth mode, diet, and atopic disorders in a longitudinal analysis of stool samples, collected from infancy through early childhood. Gastroenterology.

[42] Correa-Oliveira R, Fachi JL, Vieira A, Sato FT, Vinolo MA (2016). Regulation of immune cell function by short-chain fatty acids. Clin. Transl. Immunol..

[43] Korpela K (2018). Selective maternal seeding and environment shape the human gut microbiome. Genome Res..

[44] Koren O (2012). Host remodeling of the gut microbiome and metabolic changes during pregnancy. Cell.

[45] Sakwinska O (2017). Does the maternal vaginal microbiota play a role in seeding the microbiota of neonatal gut and nose?. Benef. Microbes.

[46] Drell T (2017). The influence of different maternal microbial communities on the development of infant gut and oral microbiota. Sci. Rep..

[47] Avershina E (2016). Transition from infant- to adult-like gut microbiota. Environ. Microbiol..

[48] Dierikx T (2021). Influence of timing of maternal antibiotic administration during caesarean section on infant microbial colonisation: A randomised controlled trial. Gut.

[49] Liu X (2021). Blautia: A new functional genus with potential probiotic properties?. Gut Microbes.

[50] Coker MO (2020). Specific class of intrapartum antibiotics relates to maturation of the infant gut microbiota: A prospective cohort study. BJOG.

[51] Enomoto A (2018). A preliminary study of the effect of room temperature incubation on phylogenetic composition of salivary microbiota. J. Oral Sci. Rehabil..

[52] Levin AM (2016). Joint effects of pregnancy, sociocultural, and environmental factors on early life gut microbiome structure and diversity. Sci. Rep..

