# Supplementary material for: Development of gut microbiota during the first 2 years of life
Source: Sci Rep. 2022 May 31;12:9080. doi: 10.1038/s41598-022-13009-3 (PMC9156670; doi:10.1038/s41598-022-13009-3)
Supplement: Supplementary file 1 — Supplementary Information. [file 41598_2022_13009_MOESM1_ESM.pdf]

SUPPLEMENTARY INFORMATION for

Development of gut microbiota during the first 2 years of life

Mona-Lisa Wernroth, Sari Peura, Anna M. Hedman, Susanne Hetty, Silvia Vicenzi, Beatrice Kennedy, Katja Fall, Bodil Svennblad, Ellika Andolf, Göran Pershagen, Jenny Theorell-Haglöw, Diem Nguyen, Sergi Sayols-Baixeras, Koen F. Dekkers, Stefan Bertilsson, Catarina Almqvist, Johan Dicksved, Tove Fall

Supplementary Figure S1. Directed Acyclic Graph of theoretical framework to investigate the effect of mode of delivery on gut microbiota in children. A directed edge (or "arrow") from one node to another represents a direct effect between these two nodes.

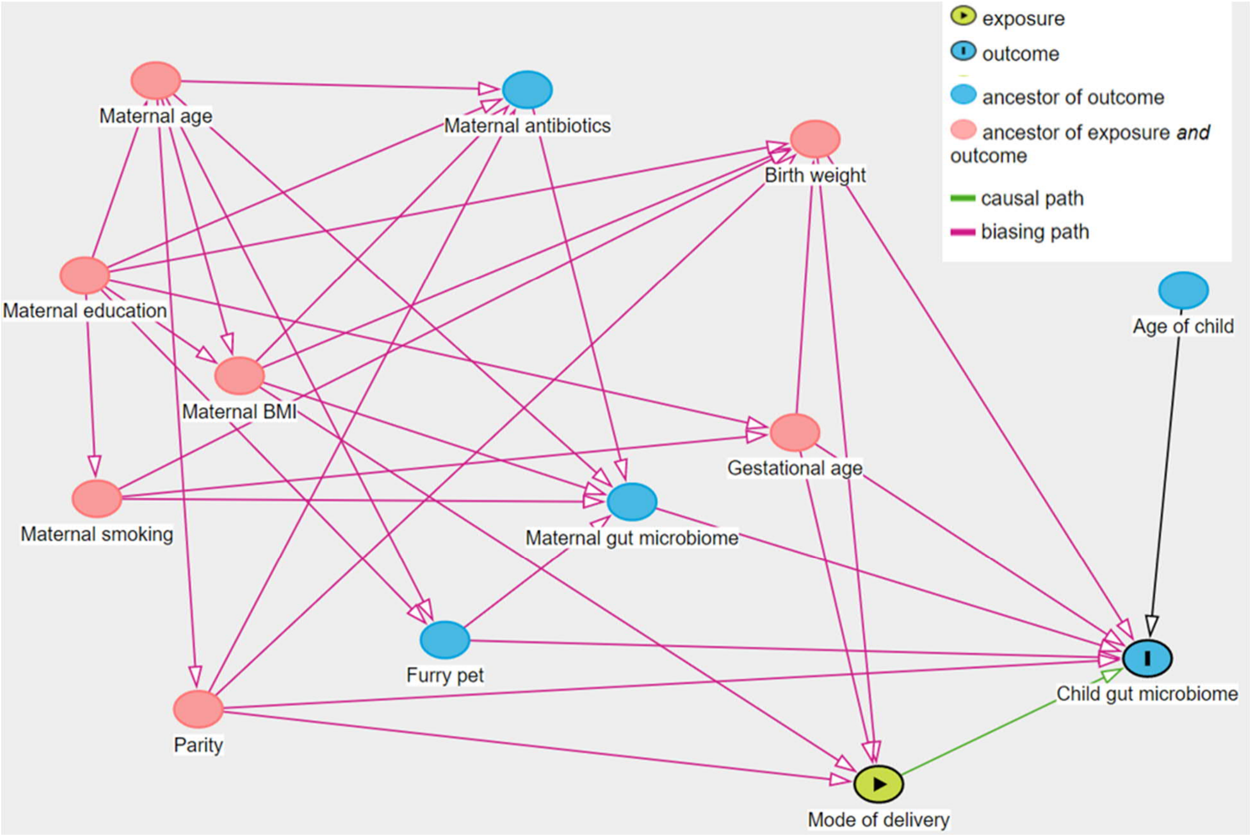

Supplementary Figure S2. Model-based predictions of the inverse Simpson index of the gut microbiota in children plotted against the age. Lines indicate mean values (solid) and 95% confidence intervals (dashed).

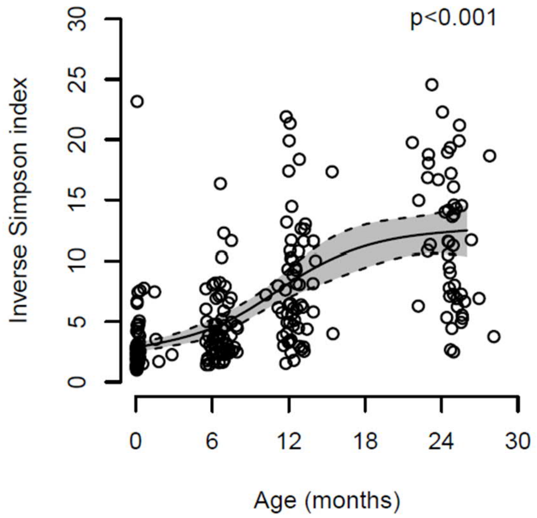

Supplementary Figure S3. Comparison of microbiota composition based on Jaccard distances. The shorter the distance between any pair of comparisons indicate greater similarity between microbial communities collected at those times. W = gestational week and m = month. Symbols represent means and error bars 95% confidence intervals. a Comparisons of gut microbiota at different sampling times, where distances between different sampling points within the same child/mother (self), and distances between unrelated children (non-self) and between unrelated mothers (non-self) are denoted. b Comparisons between gut and oral microbiota, and between children and their mothers and unrelated mothers. The distance between child and mother is based on the maternal sample at gestational week 26-28.

a

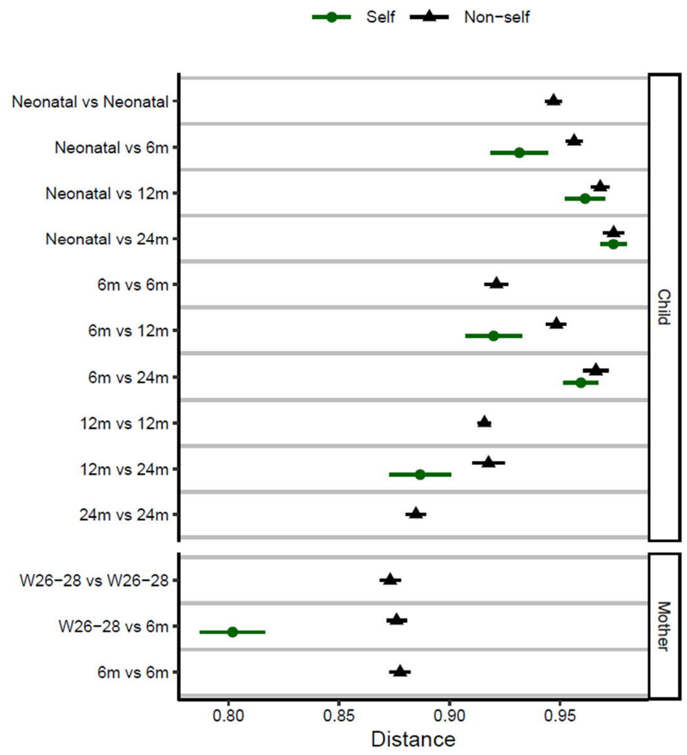

b

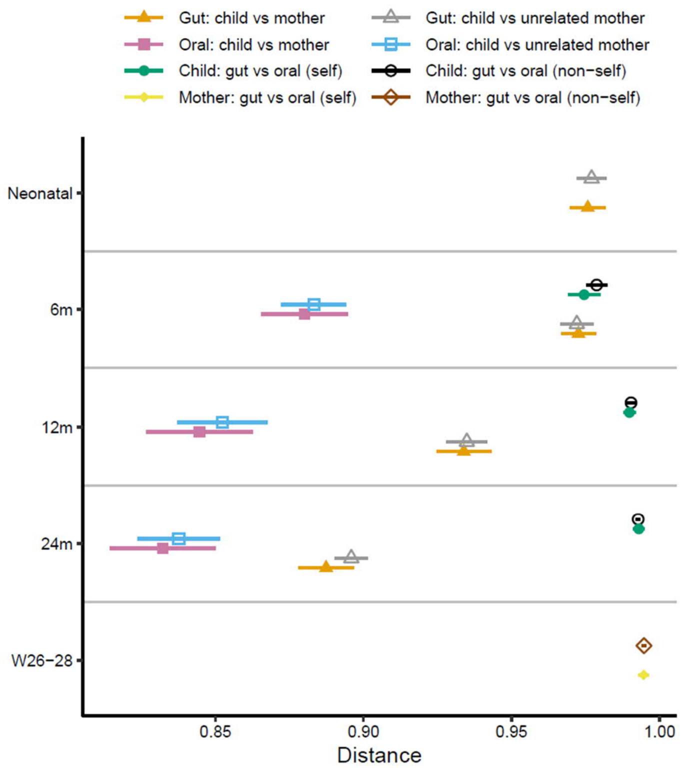

Supplementary Figure S4. Model-based predictions of relative abundance of families in the gut of children plotted against age. Lines indicate mean values (solid) and 95% confidence intervals (dashed). The y-axis shows the relative abundance in proportion. Only observations < 97th percentile are shown.

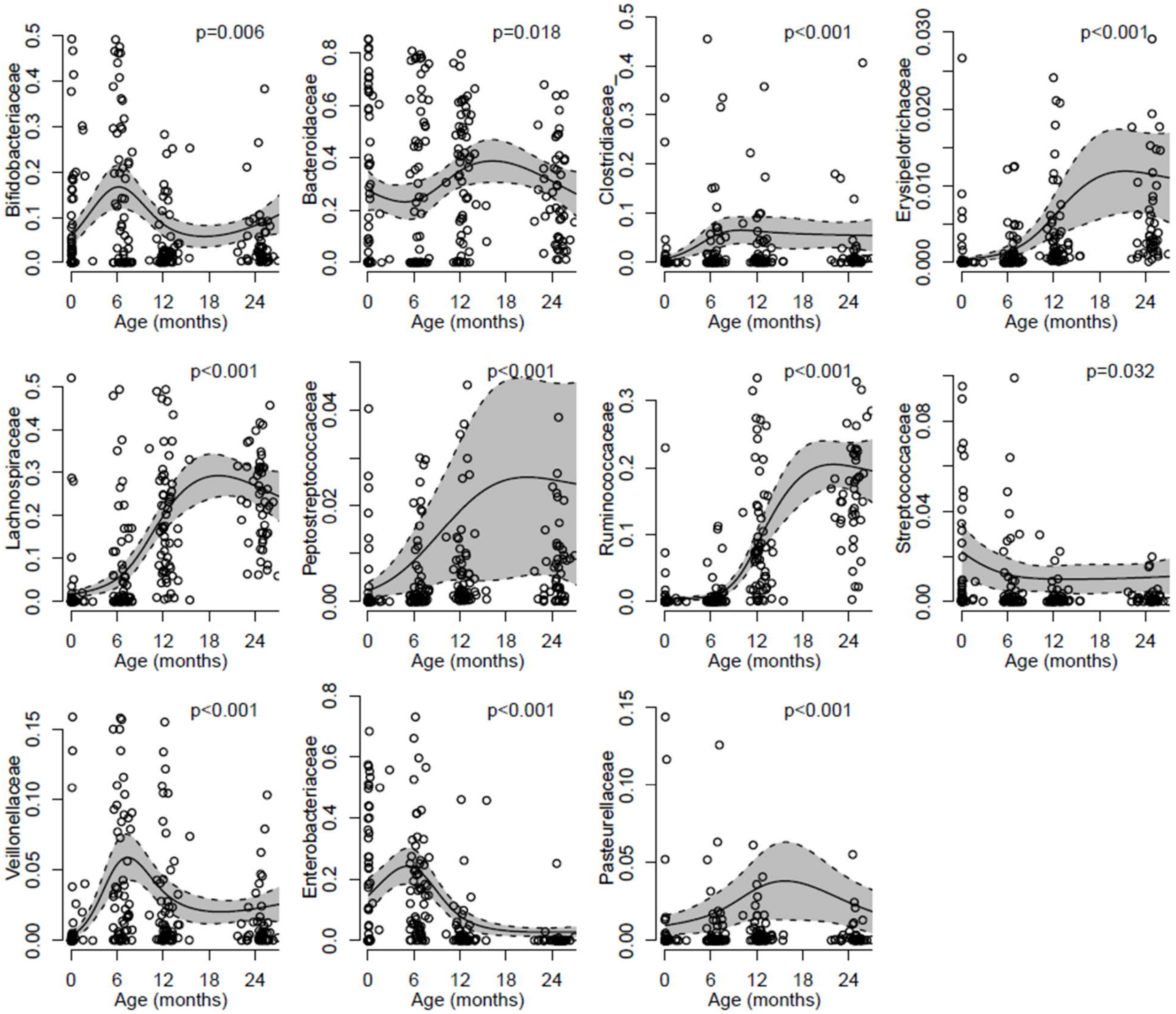

Supplementary Figure S5. Stacked bar chart of the relative distribution of operational taxonomic units (OTUs) and associated genera detected in the children's and mothers' gut microbiota. The 20 most abundant OTUs detected in the children's gut microbiota are depicted.

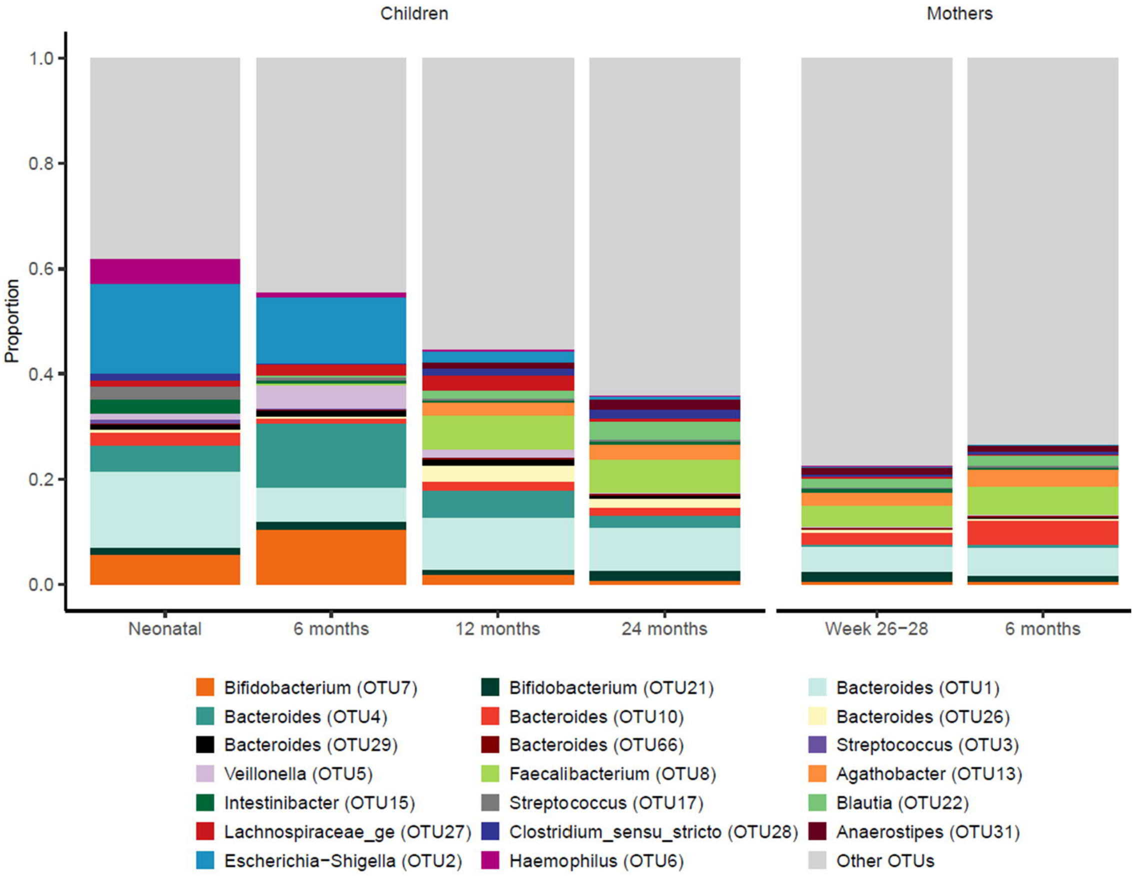

Supplementary Figure S6. Model-based predictions of relative abundance of operational taxonomic units (OTUs) in the gut of children plotted against age. Lines indicate mean values (solid) and 95% confidence intervals (dashed). The y-axis shows the relative abundance in proportion. Only observations < 97th percentile are shown.

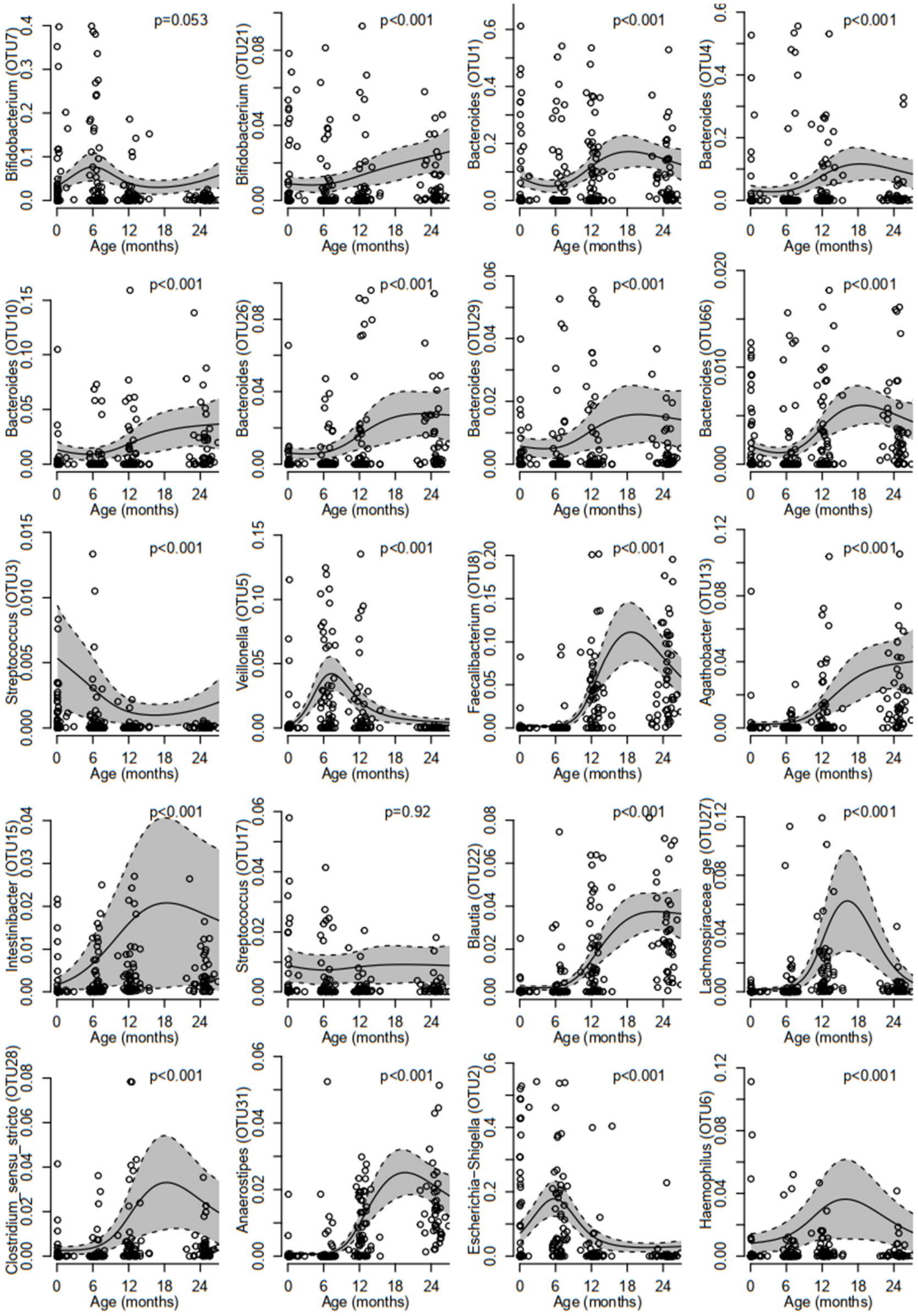

Supplementary Figure S7. Model-based predictions of relative abundance of the phylum Bacteroidetes in the gut of children plotted against age. Predictions are separated by the mothers' relative abundance of Bacteroidetes (at quartiles) at gestational week 26-28. Lines indicate mean values adjusted for potential confounders (see Table 1). All continuous adjustment variables are fixed at their median value and all categorical adjustment variables are fixed at their most common category. The y-axis shows the relative abundance in proportion.

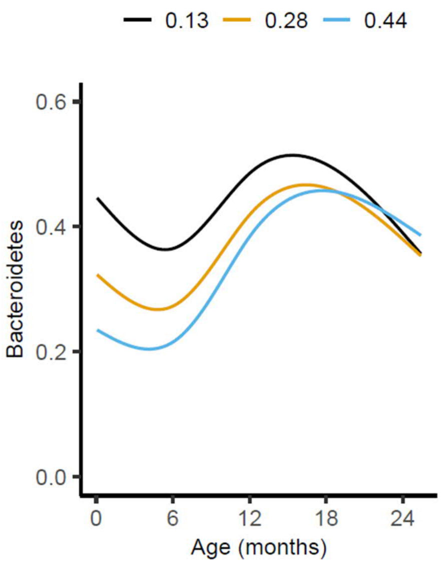

Supplementary Figure S8. Model-based predictions of relative abundance of Veillonella (operational taxonomic unit (OTU) 5) in the gut of children plotted against the mother's proportion of Veillonella (OTU5). Lines indicate mean values (solid) and 95% confidence intervals (dashed), adjusted for children's age and potential confounders (see Table 1). All continuous adjustment variables are fixed at their median value and all categorical adjustment variables are fixed at their most common category

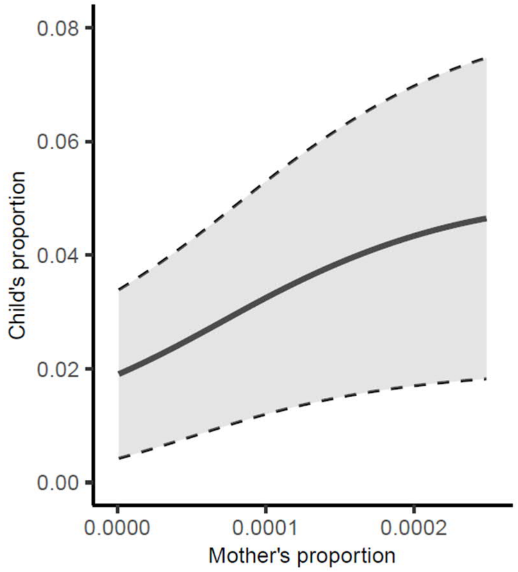

Supplementary Figure S9. Principal coordinate analysis plot (Bray-Curtis distances) with centroids (large symbols) depicting differences in overall gut microbiota composition across four time points, between children between vaginally- and caesarean-delivered children. M = month

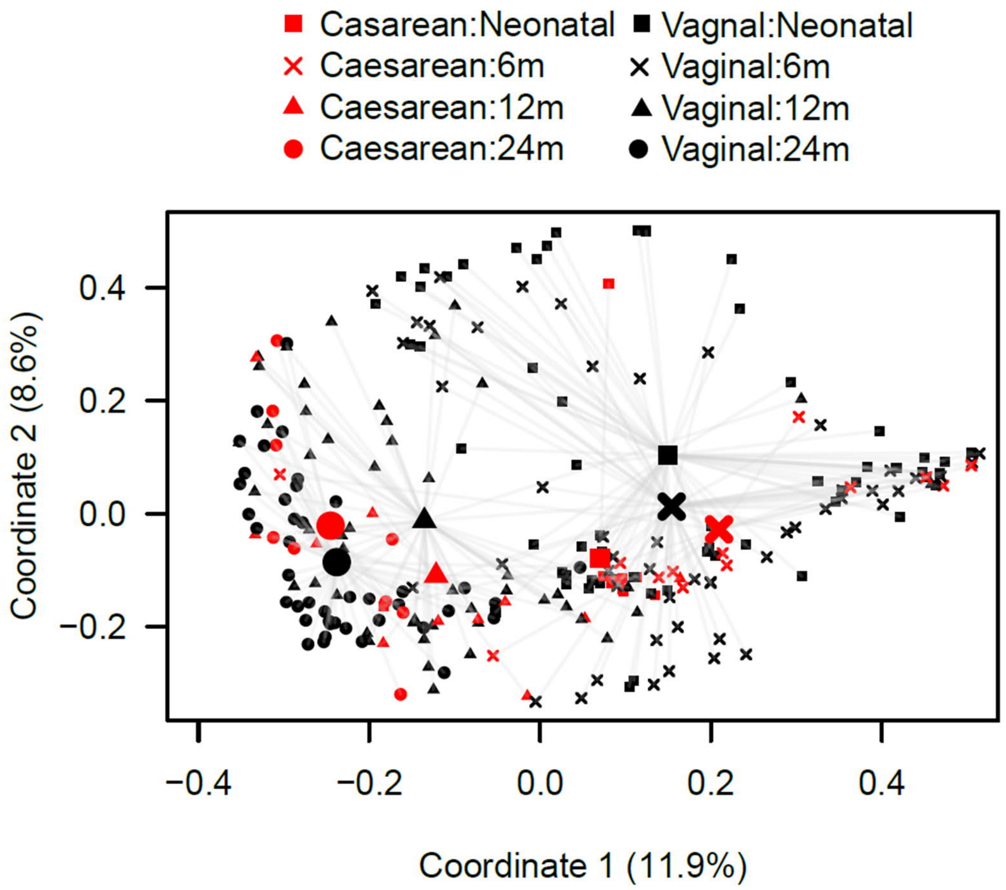

Supplementary Figure S10. Model-based predictions of relative abundance of the family Bacteroidaceae and Streptococcaceae in the gut of children by mode of delivery plotted against age. Lines indicate mean values (solid) and 95% confidence intervals (dashed), adjusted for potential confounders (see Table 1). P reflect for the interaction between mode of delivery and child age. All continuous adjustment variables are fixed at their median value and all categorical adjustment variables are fixed at their most common category. For Streptococcaceae are 3 observations > 99th percentile not shown.

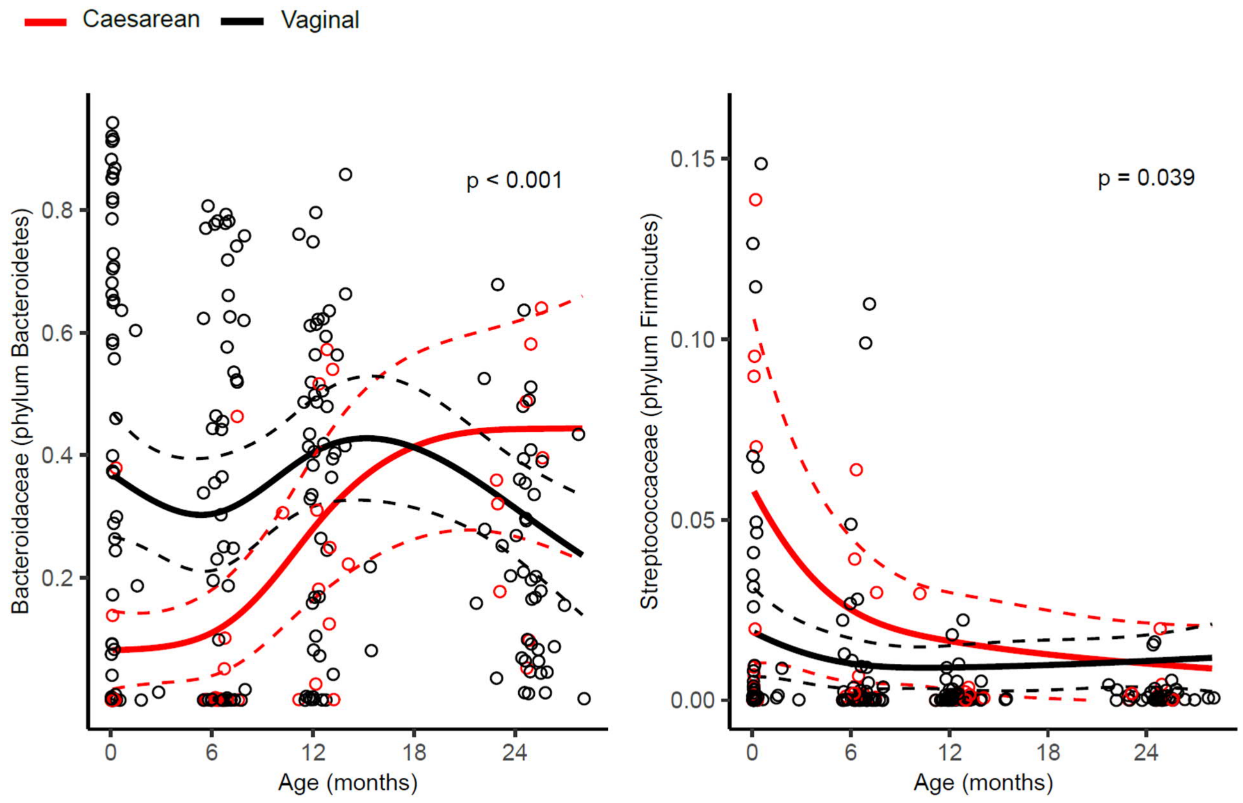

Supplementary Figure S11. Model-based predictions of relative abundance of the phylum Firmicutes and an operational taxonomic unit (OTU) belonging to the genus Streptococcus (OTU3) in the gut of children plotted against age. Predictions are separated by gestational age in days (at quartiles). Lines indicate mean values adjusted for potential confounders (see Table 1). All continuous adjustment variables are fixed at their median value and all categorical adjustment variables are fixed at their most common category. The y-axis shows the relative abundance in proportion.

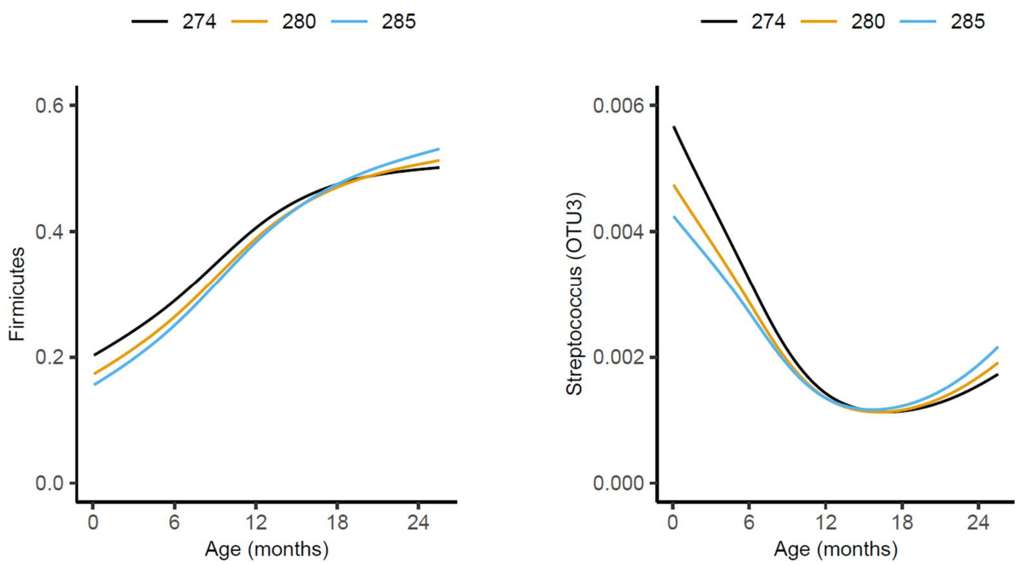

Supplementary Figure S12. Model-based prediction of relative abundance of operational taxonomic units (OTUs) belonging to the genus Bacteroides in the gut of children plotted against birth weight. Lines indicate mean values (solid) and 95% confidence intervals (dashed), adjusted for children's age and potential confounders (see Table 1). All continuous adjustment variables are fixed at their median value and all categorical adjustment variables are fixed at their most common category. The y-axis shows the relative abundance in proportion.

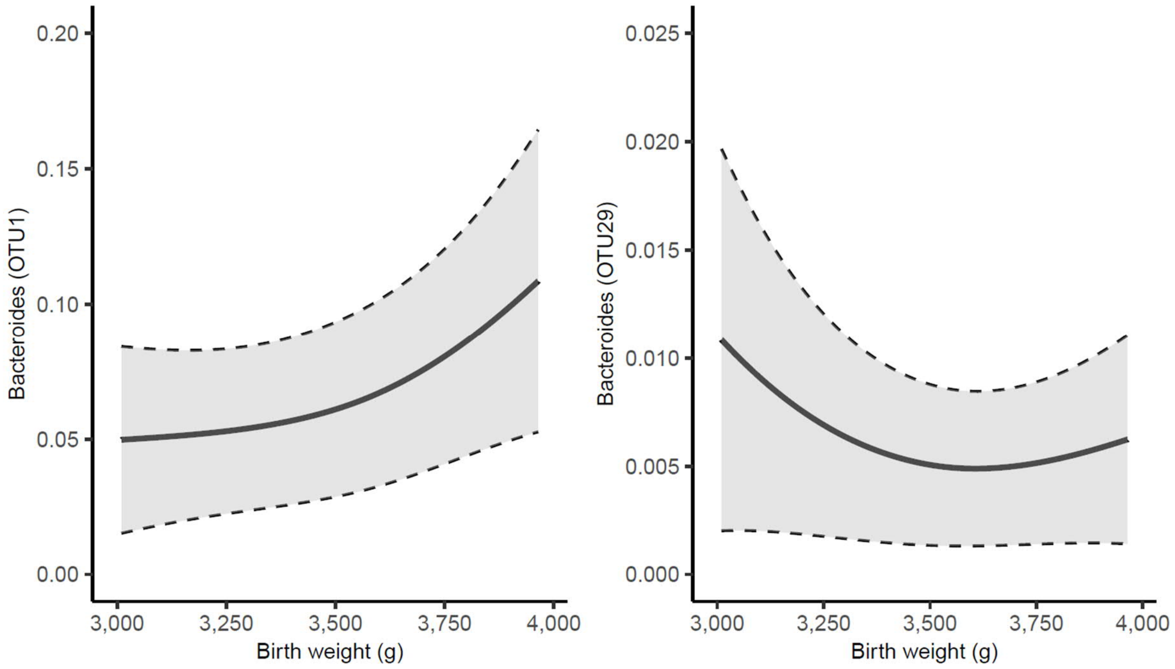

Supplementary Figure S13. Model-based prediction of relative abundance of the phylum Actinobacteria and an operational taxonomic unit (OTU) belonging to the genus Bifidobacterium (OTU21) in the gut of children by the presence of furry pets in the household, plotted against age. Lines indicate mean values (solid) and 95% confidence intervals (dashed), adjusted for potential confounders (see Table 1). All continuous adjustment variables are fixed at their median value and all categorical adjustment variables are fixed at their most common category. The y-axis shows the relative abundance in proportion. For Bifidobacterium (OTU21) five observations > 97th percentile are not shown.

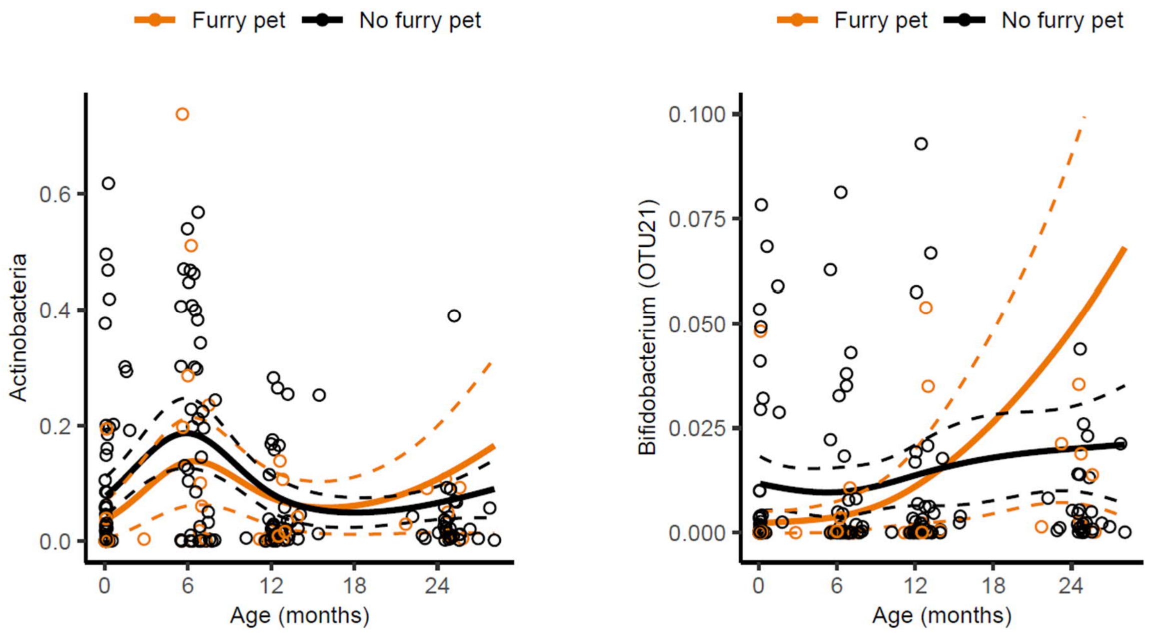

Supplementary Figure S14. Model-based prediction of relative abundance of the family Bifidobacteriaceae in the gut of children by the presence of furry pets in the household, plotted against age. Lines indicate mean values (solid) and 95% confidence intervals (dashed), adjusted for potential confounders (see Table 1). P reflect for the interaction between furry pet and child age. All continuous adjustment variables are fixed at their median value and all categorical adjustment variables are fixed at their most common category. The y-axis shows the relative abundance in proportion.

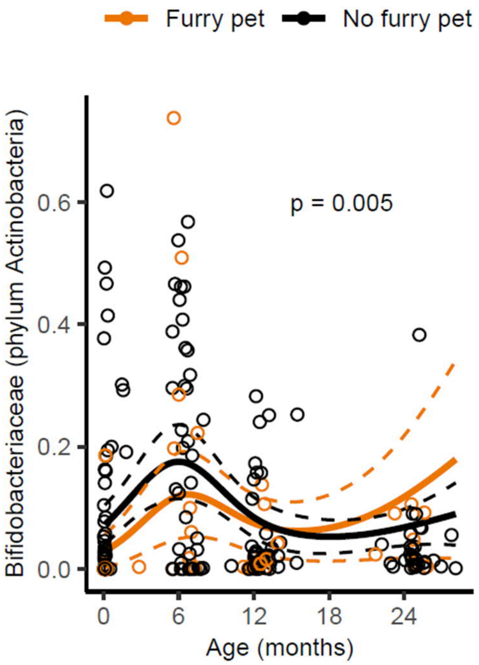

Supplementary Table S1. The 20 most common operational taxonomic units (OTUs) in the gut microbiota of children.

Phylum	Class	Order	Family	Genus	OTU number
Actinobacteria	Actinobacteria	Bifidobacteriales	Bifidobacteriaceae	Bifidobacterium	7
Actinobacteria	Actinobacteria	Bifidobacteriales	Bifidobacteriaceae	Bifidobacterium	21
Bacteroidetes	Bacteroidia	Bacteroidales	Bacteroidaceae	Bacteroides	1
Bacteroidetes	Bacteroidia	Bacteroidales	Bacteroidaceae	Bacteroides	4
Bacteroidetes	Bacteroidia	Bacteroidales	Bacteroidaceae	Bacteroides	10
Bacteroidetes	Bacteroidia	Bacteroidales	Bacteroidaceae	Bacteroides	26
Bacteroidetes	Bacteroidia	Bacteroidales	Bacteroidaceae	Bacteroides	29
Bacteroidetes	Bacteroidia	Bacteroidales	Bacteroidaceae	Bacteroides	66
Firmicutes	Bacilli	Lactobacillales	Streptococcaceae	Streptococcus	3
Firmicutes	Bacilli	Lactobacillales	Streptococcaceae	Streptococcus	17
Firmicutes	Clostridia	Clostridiales	Clostridiaceae_1	Clostridium_sensu_stricto_1	28
Firmicutes	Clostridia	Clostridiales	Lachnospiraceae	Agathobacter	13
Firmicutes	Clostridia	Clostridiales	Lachnospiraceae	Anaerostipes	31
Firmicutes	Clostridia	Clostridiales	Lachnospiraceae	Blautia	22
Firmicutes	Clostridia	Clostridiales	Lachnospiraceae	Lachnospiraceae_ge	27
Firmicutes	Clostridia	Clostridiales	Peptostreptococcaceae	Intestinibacter	15
Firmicutes	Clostridia	Clostridiales	Ruminococcaceae	Faecalibacterium	8
Firmicutes	Negativicutes	Selenomonadales	Veillonellaceae	Veillonella	5
Proteobacteria	Gammaproteobacteria	Enterobacteriales	Enterobacteriaceae	Escherichia-Shigella	2
Proteobacteria	Gammaproteobacteria	Pasteurellales	Pasteurellaceae	Haemophilus	6

Supplementary Table S2. Results from ordinal regression analyses and PERMANOVA. The FDR cut-off corresponded to a nominal p of 0.0213. OTU: operational taxonomic unit, m: month, neo: neonatal and beta: beta diversity

**Exposure**	**Outcome**	**Phylum**	**Genus**	**OTU number**	**p**	**Interaction with age**
Age	Inverse Simpson			.	<.0001	
Age	Phyla	Actinobacteria		.	0.0049	
Age	OTU	Actinobacteria	Bifidobacterium	7	0.0528	
Age	OTU	Actinobacteria	Bifidobacterium	21	<.0001	
Age	Phyla	Bacteroidetes		.	0.0153	
Age	OTU	Bacteroidetes	Bacteroides	1	<.0001	
Age	OTU	Bacteroidetes	Bacteroides	4	<.0001	
Age	OTU	Bacteroidetes	Bacteroides	10	<.0001	
Age	OTU	Bacteroidetes	Bacteroides	26	<.0001	
Age	OTU	Bacteroidetes	Bacteroides	29	<.0001	
Age	OTU	Bacteroidetes	Bacteroides	66	<.0001	
Age	Phyla	Firmicutes		.	<.0001	
Age	OTU	Firmicutes	Streptococcus	3	<.0001	
Age	OTU	Firmicutes	Veillonella	5	<.0001	
Age	OTU	Firmicutes	Faecalibacterium	8	<.0001	
Age	OTU	Firmicutes	Agathobacter	13	<.0001	
Age	OTU	Firmicutes	Intestinibacter	15	<.0001	
Age	OTU	Firmicutes	Streptococcus	17	0.9216	
Age	OTU	Firmicutes	Blautia	22	<.0001	
Age	OTU	Firmicutes	Lachnospiraceae_ge	27	<.0001	
Age	OTU	Firmicutes	Clostridium_sensu_stricto_	28	<.0001	
Age	OTU	Firmicutes	Anaerostipes	31	<.0001	
Age	Phyla	Proteobacteria		.	<.0001	
Age	OTU	Proteobacteria	Escherichia-Shigella	2	<.0001	
Age	OTU	Proteobacteria	Haemophilus	6	0.0001	
Maternal W26-28	Inverse Simpson			.	0.0849	No
Maternal W26-28	Phyla	Actinobacteria		.	0.2691	No
Maternal W26-28	OTU	Actinobacteria	Bifidobacterium	7	0.0160[‡]	Yes
Maternal W26-28	OTU	Actinobacteria	Bifidobacterium	21	0.0270	Yes
Maternal W26-28	Phyla	Bacteroidetes		.	0.0056	Yes
Maternal W26-28	OTU	Bacteroidetes	Bacteroides	1	0.6707	No

Exposure	Outcome	Phylum	Genus	OTU number	p	Interaction with age
Maternal W26-28	OTU	Bacteroidetes	Bacteroides	4	0.3301	No
Maternal W26-28	OTU	Bacteroidetes	Bacteroides	10	0.2625	No
Maternal W26-28	OTU	Bacteroidetes	Bacteroides	26	0.1274	No
Maternal W26-28	OTU	Bacteroidetes	Bacteroides	29	0.3754	No
Maternal W26-28	OTU	Bacteroidetes	Bacteroides	66	0.4180	No
Maternal W26-28	Phyla	Firmicutes		.	0.3952	No
Maternal W26-28	OTU	Firmicutes	Streptococcus	3	0.1977	No
Maternal W26-28	OTU	Firmicutes	Veillonella	5	0.0012	No
Maternal W26-28	OTU	Firmicutes	Faecalibacterium	8	0.4082	No
Maternal W26-28	OTU	Firmicutes	Agathobacter	13	0.9336	No
Maternal W26-28	OTU	Firmicutes	Intestinibacter	15	0.1032	No
Maternal W26-28	OTU	Firmicutes	Streptococcus	17	0.0094‡	Yes
Maternal W26-28	OTU	Firmicutes	Blautia	22	0.8490	No
Maternal W26-28	OTU	Firmicutes	Lachnospiraceae_ge	27	0.0170‡	Yes
Maternal W26-28	OTU	Firmicutes	Clostridium_sensu_stricto_	28	0.3917	No
Maternal W26-28	OTU	Firmicutes	Anaerostipes	31	0.3798	No
Maternal W26-28	Phyla	Proteobacteria		.	0.0258	Yes
Maternal W26-28	OTU	Proteobacteria	Escherichia-Shigella	2	0.1577	No
Maternal W26-28	OTU	Proteobacteria	Haemophilus	6	0.1968	No
Mode of delivery	Beta Neo.			.	0.0065	
Mode of delivery	Beta 6M			.	0.1269	
Mode of delivery	Beta 12M			.	0.7686	
Mode of delivery	Beta 24M			.	0.7246	
Mode of delivery	Inverse Simpson			.	0.4302	No
Mode of delivery	Phyla	Actinobacteria		.	0.5895	No
Mode of delivery	OTU	Actinobacteria	Bifidobacterium	7	0.4765	No
Mode of delivery	OTU	Actinobacteria	Bifidobacterium	21	0.1357	No
Mode of delivery	Phyla	Bacteroidetes		.	<.0001	Yes
Mode of delivery	OTU	Bacteroidetes	Bacteroides	1	0.0151	Yes
Mode of delivery	OTU	Bacteroidetes	Bacteroides	4	0.9487	No
Mode of delivery	OTU	Bacteroidetes	Bacteroides	10	0.0372	No
Mode of delivery	OTU	Bacteroidetes	Bacteroides	26	0.0331	No
Mode of delivery	OTU	Bacteroidetes	Bacteroides	29	0.0085	No
Mode of delivery	OTU	Bacteroidetes	Bacteroides	66	0.0062	Yes
Mode of delivery	Phyla	Firmicutes		.	<.0001	Yes

Exposure	Outcome	Phylum	Genus	OTU number	p	Interaction with age
Mode of delivery	OTU	Firmicutes	Streptococcus	3	0.0162	Yes
Mode of delivery	OTU	Firmicutes	Veillonella	5	0.0094	No
Mode of delivery	OTU	Firmicutes	Faecalibacterium	8	0.0029	No
Mode of delivery	OTU	Firmicutes	Agathobacter	13	0.7241	No
Mode of delivery	OTU	Firmicutes	Intestinibacter	15	0.0713	No
Mode of delivery	OTU	Firmicutes	Streptococcus	17	0.0164	No
Mode of delivery	OTU	Firmicutes	Blautia	22	0.4449	No
Mode of delivery	OTU	Firmicutes	Lachnospiraceae_ge	27	0.0941	No
Mode of delivery	OTU	Firmicutes	Clostridium_sensu_stricto_	28	0.9486	No
Mode of delivery	OTU	Firmicutes	Anaerostipes	31	0.0144‡	Yes
Mode of delivery	Phyla	Proteobacteria		.	0.2723	No
Mode of delivery	OTU	Proteobacteria	Escherichia-Shigella	2	0.0008	Yes
Mode of delivery	OTU	Proteobacteria	Haemophilus	6	0.2934	No
Parity	Beta Neo.			.	0.3748	
Parity	Beta 6M			.	0.5152	
Parity	Beta 12M			.	0.1744	
Parity	Beta 24M			.	0.1604	
Parity	Inverse Simpson			.	0.1720	No
Parity	Phyla	Actinobacteria		.	0.1540	No
Parity	OTU	Actinobacteria	Bifidobacterium	7	0.1880	No
Parity	OTU	Actinobacteria	Bifidobacterium	21	0.0226	No
Parity	Phyla	Bacteroidetes		.	0.9676	No
Parity	OTU	Bacteroidetes	Bacteroides	1	0.3157	No
Parity	OTU	Bacteroidetes	Bacteroides	4	0.0421	Yes
Parity	OTU	Bacteroidetes	Bacteroides	10	0.3514	No
Parity	OTU	Bacteroidetes	Bacteroides	26	0.0617	No
Parity	OTU	Bacteroidetes	Bacteroides	29	0.3496	No
Parity	OTU	Bacteroidetes	Bacteroides	66	0.8153	No
Parity	Phyla	Firmicutes		.	0.9991	No
Parity	OTU	Firmicutes	Streptococcus	3	0.1621	No
Parity	OTU	Firmicutes	Veillonella	5	0.2491	No
Parity	OTU	Firmicutes	Faecalibacterium	8	0.5068	No
Parity	OTU	Firmicutes	Agathobacter	13	0.6753	No
Parity	OTU	Firmicutes	Intestinibacter	15	0.6297	No
Parity	OTU	Firmicutes	Streptococcus	17	0.0480	Yes

Exposure	Outcome	Phylum	Genus	OTU number	p	Interaction with age
Parity	OTU	Firmicutes	Blautia	22	0.6606	No
Parity	OTU	Firmicutes	Lachnospiraceae_ge	27	0.8005	No
Parity	OTU	Firmicutes	Clostridium_sensu_stricto_	28	0.0322	No
Parity	OTU	Firmicutes	Anaerostipes	31	0.1825	No
Parity	Phyla	Proteobacteria		.	0.4039	No
Parity	OTU	Proteobacteria	Escherichia-Shigella	2	0.8329	No
Parity	OTU	Proteobacteria	Haemophilus	6	0.0046	No
Gestational age	Beta Neo.			.	0.1354	
Gestational age	Beta 6M			.	0.4838	
Gestational age	Beta 12M			.	0.4383	
Gestational age	Beta 24M			.	0.2539	
Gestational age	Inverse Simpson			.	0.8197	No
Gestational age	Phyla	Actinobacteria		.	0.6981	No
Gestational age	OTU	Actinobacteria	Bifidobacterium	7	0.4557	No
Gestational age	OTU	Actinobacteria	Bifidobacterium	21	0.5600	No
Gestational age	Phyla	Bacteroidetes		.	0.0370	Yes
Gestational age	OTU	Bacteroidetes	Bacteroides	1	0.1545	No
Gestational age	OTU	Bacteroidetes	Bacteroides	4	0.1378	No
Gestational age	OTU	Bacteroidetes	Bacteroides	10	0.9830	No
Gestational age	OTU	Bacteroidetes	Bacteroides	26	0.6707	No
Gestational age	OTU	Bacteroidetes	Bacteroides	29	0.5451	No
Gestational age	OTU	Bacteroidetes	Bacteroides	66	0.1957	No
Gestational age	Phyla	Firmicutes		.	0.0158	Yes
Gestational age	OTU	Firmicutes	Streptococcus	3	0.0213	Yes
Gestational age	OTU	Firmicutes	Veillonella	5	0.1530	No
Gestational age	OTU	Firmicutes	Faecalibacterium	8	0.6364	No
Gestational age	OTU	Firmicutes	Agathobacter	13	0.1069	No
Gestational age	OTU	Firmicutes	Intestinibacter	15	0.4131	No
Gestational age	OTU	Firmicutes	Streptococcus	17	0.1368	No
Gestational age	OTU	Firmicutes	Blautia	22	0.1807	No
Gestational age	OTU	Firmicutes	Lachnospiraceae_ge	27	0.0541	No
Gestational age	OTU	Firmicutes	Clostridium_sensu_stricto_	28	0.0005‡	No
Gestational age	OTU	Firmicutes	Anaerostipes	31	0.1156	No
Gestational age	Phyla	Proteobacteria		.	0.5605	No
Gestational age	OTU	Proteobacteria	Escherichia-Shigella	2	0.0004‡	Yes

Exposure	Outcome	Phylum	Genus	OTU number	p	Interaction with age
Gestational age	OTU	Proteobacteria	Haemophilus	6	0.2063	No
Birth weight	Beta Neo.			.	0.1744	
Birth weight	Beta 6M			.	0.6067	
Birth weight	Beta 12M			.	0.0200	
Birth weight	Beta 24M			.	0.9390	
Birth weight	Inverse Simpson			.	0.8412	No
Birth weight	Phyla	Actinobacteria		.	0.6559	No
Birth weight	OTU	Actinobacteria	Bifidobacterium	7	0.3328	No
Birth weight	OTU	Actinobacteria	Bifidobacterium	21	0.6611	No
Birth weight	Phyla	Bacteroidetes		.	0.5653	No
Birth weight	OTU	Bacteroidetes	Bacteroides	1	0.0070	No
Birth weight	OTU	Bacteroidetes	Bacteroides	4	0.8128	No
Birth weight	OTU	Bacteroidetes	Bacteroides	10	0.9301	No
Birth weight	OTU	Bacteroidetes	Bacteroides	26	0.2854	No
Birth weight	OTU	Bacteroidetes	Bacteroides	29	0.0130	No
Birth weight	OTU	Bacteroidetes	Bacteroides	66	0.0399	No
Birth weight	Phyla	Firmicutes		.	0.8592	No
Birth weight	OTU	Firmicutes	Streptococcus	3	0.6983	No
Birth weight	OTU	Firmicutes	Veillonella	5	0.6706	No
Birth weight	OTU	Firmicutes	Faecalibacterium	8	0.7032	No
Birth weight	OTU	Firmicutes	Agathobacter	13	0.8801	No
Birth weight	OTU	Firmicutes	Intestinibacter	15	0.0515	No
Birth weight	OTU	Firmicutes	Streptococcus	17	0.2552	No
Birth weight	OTU	Firmicutes	Blautia	22	0.0303	Yes
Birth weight	OTU	Firmicutes	Lachnospiraceae_ge	27	0.1898	No
Birth weight	OTU	Firmicutes	Clostridium_sensu_stricto_	28	0.7989	No
Birth weight	OTU	Firmicutes	Anaerostipes	31	0.7833	No
Birth weight	Phyla	Proteobacteria		.	0.1101	No
Birth weight	OTU	Proteobacteria	Escherichia-Shigella	2	0.0036[‡]	Yes
Birth weight	OTU	Proteobacteria	Haemophilus	6	0.0405	Yes
Antibiotics (prenatal)	Beta Neo.			.	0.1004	
Antibiotics (prenatal)	Beta 6M			.	0.5602	
Antibiotics (prenatal)	Beta 12M			.	0.1704	
Antibiotics (prenatal)	Beta 24M			.	0.4723	
Antibiotics (prenatal)	Inverse Simpson			.	0.1508	No

Exposure	Outcome	Phylum	Genus	OTU number	p	Interaction with age
Antibiotics (prenatal)	Phyla	Actinobacteria		.	0.7551	No
Antibiotics (prenatal)	OTU	Actinobacteria	Bifidobacterium	7	0.6820	No
Antibiotics (prenatal)	OTU	Actinobacteria	Bifidobacterium	21	0.8224	No
Antibiotics (prenatal)	Phyla	Bacteroidetes		.	0.9674	No
Antibiotics (prenatal)	OTU	Bacteroidetes	Bacteroides	1	0.1125	No
Antibiotics (prenatal)	OTU	Bacteroidetes	Bacteroides	4	0.8298	No
Antibiotics (prenatal)	OTU	Bacteroidetes	Bacteroides	10	0.8690	No
Antibiotics (prenatal)	OTU	Bacteroidetes	Bacteroides	26	0.9869	No
Antibiotics (prenatal)	OTU	Bacteroidetes	Bacteroides	29	0.8684	No
Antibiotics (prenatal)	OTU	Bacteroidetes	Bacteroides	66	0.4508	No
Antibiotics (prenatal)	Phyla	Firmicutes		.	0.1103	No
Antibiotics (prenatal)	OTU	Firmicutes	Streptococcus	3	0.8583	No
Antibiotics (prenatal)	OTU	Firmicutes	Veillonella	5	0.0910	No
Antibiotics (prenatal)	OTU	Firmicutes	Faecalibacterium	8	0.0163‡	Yes
Antibiotics (prenatal)	OTU	Firmicutes	Agathobacter	13	0.1060	No
Antibiotics (prenatal)	OTU	Firmicutes	Intestinibacter	15	0.1466	No
Antibiotics (prenatal)	OTU	Firmicutes	Streptococcus	17	0.4135	No
Antibiotics (prenatal)	OTU	Firmicutes	Blautia	22	0.0161	No
Antibiotics (prenatal)	OTU	Firmicutes	Lachnospiraceae_ge	27	0.5490	No
Antibiotics (prenatal)	OTU	Firmicutes	Clostridium_sensu_stricto_	28	0.3949	No
Antibiotics (prenatal)	OTU	Firmicutes	Anaerostipes	31	0.0700	No
Antibiotics (prenatal)	Phyla	Proteobacteria		.	0.1111	No
Antibiotics (prenatal)	OTU	Proteobacteria	Escherichia-Shigella	2	0.0467	Yes
Antibiotics (prenatal)	OTU	Proteobacteria	Haemophilus	6	0.9159	No
Antibiotics (ever-use)	Beta 24M			.	0.3503	
Antibiotics (ever-use)	Inverse Simpson			.	0.8050	
Antibiotics (ever-use)	Phyla	Actinobacteria		.	0.4060	
Antibiotics (ever-use)	OTU	Actinobacteria	Bifidobacterium	7	0.5908	
Antibiotics (ever-use)	OTU	Actinobacteria	Bifidobacterium	21	0.4960	
Antibiotics (ever-use)	Phyla	Bacteroidetes		.	0.5604	
Antibiotics (ever-use)	OTU	Bacteroidetes	Bacteroides	1	0.1678	
Antibiotics (ever-use)	OTU	Bacteroidetes	Bacteroides	4	0.6575	
Antibiotics (ever-use)	OTU	Bacteroidetes	Bacteroides	10	0.2769	
Antibiotics (ever-use)	OTU	Bacteroidetes	Bacteroides	26	0.2141	
Antibiotics (ever-use)	OTU	Bacteroidetes	Bacteroides	29	0.1673	

Exposure	Outcome	Phylum	Genus	OTU number	p	Interaction with age
Antibiotics (ever-use)	OTU	Bacteroidetes	Bacteroides	66	0.8326	
Antibiotics (ever-use)	Phyla	Firmicutes		.	0.9138	
Antibiotics (ever-use)	OTU	Firmicutes	Streptococcus	3	0.1281	
Antibiotics (ever-use)	OTU	Firmicutes	Veillonella	5	0.2359	
Antibiotics (ever-use)	OTU	Firmicutes	Faecalibacterium	8	0.7881	
Antibiotics (ever-use)	OTU	Firmicutes	Agathobacter	13	0.0212[‡]	
Antibiotics (ever-use)	OTU	Firmicutes	Intestinibacter	15	0.9617	
Antibiotics (ever-use)	OTU	Firmicutes	Streptococcus	17	0.8970	
Antibiotics (ever-use)	OTU	Firmicutes	Blautia	22	0.7991	
Antibiotics (ever-use)	OTU	Firmicutes	Lachnospiraceae_ge	27	0.8822	
Antibiotics (ever-use)	OTU	Firmicutes	Clostridium_sensu_stricto_	28	0.3475	
Antibiotics (ever-use)	OTU	Firmicutes	Anaerostipes	31	0.9381	
Antibiotics (ever-use)	Phyla	Proteobacteria		.	0.7159	
Antibiotics (ever-use)	OTU	Proteobacteria	Escherichia-Shigella	2	0.1865	
Antibiotics (ever-use)	OTU	Proteobacteria	Haemophilus	6	0.1429	
Furry pet	Beta Neo.			.	0.7776	
Furry pet	Beta 6M			.	0.7816	
Furry pet	Beta 12M			.	0.7651	
Furry pet	Beta 24M			.	0.6677	
Furry pet	Inverse Simpson			.	0.6015	No
Furry pet	Phyla	Actinobacteria		.	0.0120	Yes
Furry pet	OTU	Actinobacteria	Bifidobacterium	7	0.0421	Yes
Furry pet	OTU	Actinobacteria	Bifidobacterium	21	0.0003	Yes
Furry pet	Phyla	Bacteroidetes		.	0.0904	No
Furry pet	OTU	Bacteroidetes	Bacteroides	1	0.9471	No
Furry pet	OTU	Bacteroidetes	Bacteroides	4	0.8425	No
Furry pet	OTU	Bacteroidetes	Bacteroides	10	0.0937	No
Furry pet	OTU	Bacteroidetes	Bacteroides	26	0.4678	No
Furry pet	OTU	Bacteroidetes	Bacteroides	29	0.8140	No
Furry pet	OTU	Bacteroidetes	Bacteroides	66	0.6792	No
Furry pet	Phyla	Firmicutes		.	0.2571	No
Furry pet	OTU	Firmicutes	Streptococcus	3	0.7621	No
Furry pet	OTU	Firmicutes	Veillonella	5	0.7193	No
Furry pet	OTU	Firmicutes	Faecalibacterium	8	0.7408	No
Furry pet	OTU	Firmicutes	Agathobacter	13	0.0243	Yes

Exposure	Outcome	Phylum	Genus	OTU number	p	Interaction with age
Furry pet	OTU	Firmicutes	Intestinibacter	15	0.3986	No
Furry pet	OTU	Firmicutes	Streptococcus	17	0.7647	No
Furry pet	OTU	Firmicutes	Blautia	22	0.9556	No
Furry pet	OTU	Firmicutes	Lachnospiraceae_ge	27	0.8911	No
Furry pet	OTU	Firmicutes	Clostridium_sensu_stricto_	28	0.8895	No
Furry pet	OTU	Firmicutes	Anaerostipes	31	0.3786	No
Furry pet	Phyla	Proteobacteria		.	0.6914	No
Furry pet	OTU	Proteobacteria	Escherichia-Shigella	2	0.8273	No
Furry pet	OTU	Proteobacteria	Haemophilus	6	0.7455	No

‡Exclusion of one influential observation yielded a p >0.05.

Supplementary Table S3. Comparison of microbiota composition based on Bray-Curtis and Jaccard distances. The shorter the distance between any pair of comparisons indicate greater similarity between microbial communities collected at those times. Comparisons of gut microbiota at different sampling times, where distances between different sampling points within the same child/mother (self), and distances between unrelated children (non-self) and between unrelated mothers (non-self) are presented. The Bray-Curtis distances are also depicted in Figure 3a. W = gestational week, m = month and CI Confidence Interval.

			**Bray-Curtis Mean (95% CI)**	**Jaccard Mean (95% CI)**
Child	Neonatal vs Neonatal	Non-self	0.887 (0.872-0.902)	0.947 (0.943-0.951)
Child	Neonatal vs 6m	Non-self	0.894 (0.880-0.909)	0.956 (0.953-0.960)
Child	Neonatal vs 6m	Self	0.846 (0.802-0.891)	0.932 (0.919-0.945)
Child	Neonatal vs 12m	Non-self	0.933 (0.920-0.946)	0.968 (0.964-0.972)
Child	Neonatal vs 12m	Self	0.910 (0.870-0.949)	0.961 (0.952-0.970)
Child	Neonatal vs 24m	Non-self	0.948 (0.935-0.960)	0.974 (0.969-0.979)
Child	Neonatal vs 24m	Self	0.942 (0.914-0.971)	0.974 (0.968-0.980)
Child	6m vs 6m	Non-self	0.841 (0.828-0.854)	0.921 (0.916-0.927)
Child	6m vs 12m	Non-self	0.908 (0.895-0.920)	0.948 (0.944-0.953)
Child	6m vs 12m	Self	0.832 (0.794-0.869)	0.920 (0.907-0.933)
Child	6m vs 24m	Non-self	0.935 (0.920-0.949)	0.966 (0.961-0.972)
Child	6m vs 24m	Self	0.918 (0.894-0.942)	0.959 (0.952-0.967)
Child	12m vs 12m	Non-self	0.840 (0.827-0.853)	0.916 (0.913-0.919)
Child	12m vs 24m	Non-self	0.831 (0.812-0.849)	0.918 (0.910-0.925)
Child	12m vs 24m	Self	0.754 (0.710-0.797)	0.887 (0.873-0.901)
Child	24m vs 24m	Non-self	0.768 (0.754-0.783)	0.885 (0.880-0.889)
Mother	W26-28 vs W26-28	Non-self	0.756 (0.742-0.771)	0.873 (0.868-0.878)
Mother	W26-28 vs 6m	Non-self	0.759 (0.745-0.773)	0.876 (0.872-0.881)
Mother	W26-28 vs 6m	Self	0.607 (0.572-0.642)	0.802 (0.787-0.817)
Mother	6m vs 6m	Non-self	0.758 (0.744-0.771)	0.878 (0.873-0.882)

Supplementary Table S4. Comparison of microbiota composition based on Bray-Curtis and Jaccard distances. The shorter the distance between any pair of comparisons indicate greater similarity between microbial communities collected at those times. Comparisons between gut and oral microbiota, and between children and their mothers and unrelated mothers. The distance between child and mother is based on the maternal sample at gestational week. The Bray-Curtis distances are also depicted in Figure 3b. W = gestational week, m = month and CI Confidence Interval.

		**Bray-Curtis Mean (95% CI)**	**Jaccard Mean (95% CI)**
Gut: child vs mother	Birth	0.955 (0.937-0.974)	0.976 (0.970-0.982)
Gut: child vs mother	6m	0.950 (0.933-0.968)	0.973 (0.967-0.978)
Gut: child vs mother	12m	0.862 (0.836-0.888)	0.934 (0.925-0.943)
Gut: child vs mother	24m	0.769 (0.741-0.797)	0.887 (0.878-0.897)
Gut: child vs unrelated mother	Neonatal	0.963 (0.953-0.973)	0.977 (0.972-0.982)
Gut: child vs unrelated mother	6m	0.953 (0.941-0.965)	0.972 (0.967-0.978)
Gut: child vs unrelated mother	12m	0.869 (0.852-0.885)	0.935 (0.928-0.942)
Gut: child vs unrelated mother	24m	0.793 (0.779-0.806)	0.896 (0.890-0.901)
Oral: child vs mother	6m	0.682 (0.636-0.729)	0.880 (0.865-0.895)
Oral: child vs mother	12m	0.644 (0.592-0.696)	0.845 (0.827-0.863)
Oral: child vs mother	24m	0.639 (0.587-0.691)	0.832 (0.814-0.850)
Oral: child vs unrelated mother	6m	0.687 (0.658-0.716)	0.883 (0.872-0.894)
Oral: child vs unrelated mother	12m	0.671 (0.629-0.713)	0.852 (0.837-0.868)
Oral: child vs unrelated mother	24m	0.665 (0.626-0.705)	0.838 (0.824-0.852)
Child: gut vs oral (self)	6m	0.954 (0.932-0.977)	0.975 (0.969-0.980)
Child: gut vs oral (self)	12m	0.987 (0.981-0.994)	0.990 (0.988-0.992)
Child: gut vs oral (self)	24m	0.996 (0.994-0.998)	0.993 (0.991-0.995)
Child: gut vs oral (non-self)	6m	0.963 (0.954-0.972)	0.979 (0.975-0.982)
Child: gut vs oral (non-self)	12m	0.987 (0.983-0.991)	0.990 (0.989-0.992)
Child: gut vs oral (non-self)	24m	0.996 (0.994-0.997)	0.993 (0.992-0.994)
Mother: gut vs oral (self)	W26-28	0.994 (0.991-0.997)	0.995 (0.993-0.996)
Mother: gut vs oral (non-self)	W26-28	0.994 (0.992-0.997)	0.995 (0.994-0.995)

Supplementary Table S5. P (t-test) from comparisons of the Bray-Curtis distances depicted in Figure 3a. W = gestational week, m = month, Neo=Neonatal.

			Child											
			Neo vs Neo	Neo vs 6m	Neo vs 6m	Neo vs 12m	Neo vs 12m	Neo vs 24m	Neo vs 24m	6m vs 6m	6m vs 12m	6m vs 12m	6m vs 24m	6m vs 24m
			Non-self	Non-self	Self	Non-self	Self	Non-self	Self	Non-self	Non-self	Self	Non-self	Self
Child	Neo vs Neo	Non-self		0.461	0.087	<0.001	0.278	<0.001	0.001	<0.001	0.032	0.007	<0.001	0.028
	Neo vs 6m	Non-self	0.461		0.042	<0.001	0.464	<0.001	0.004	<0.001	0.157	0.002	<0.001	0.088
	Neo vs 6m	Self	0.087	0.042		<0.001	0.034	<0.001	<0.001	0.816	0.010	0.615	<0.001	0.005
	Neo vs 12m	Non-self	<0.001	<0.001	<0.001		0.269	0.096	0.550	<0.001	0.006	<0.001	0.854	0.293
	Neo vs 12m	Self	0.278	0.464	0.034	0.269		0.069	0.184	0.001	0.925	0.005	0.240	0.707
	Neo vs 24m	Non-self	<0.001	<0.001	<0.001	0.096	0.069		0.715	<0.001	<0.001	<0.001	0.171	0.033
	Neo vs 24m	Self	0.001	0.004	<0.001	0.550	0.184	0.715		<0.001	0.031	<0.001	0.637	0.204
	6m vs 6m	Non-self	<0.001	<0.001	0.816	<0.001	0.001	<0.001	<0.001		<0.001	0.641	<0.001	<0.001
	6m vs 12m	Non-self	0.032	0.157	0.010	0.006	0.925	<0.001	0.031	<0.001		<0.001	0.006	0.434
	6m vs 12m	Self	0.007	0.002	0.615	<0.001	0.005	<0.001	<0.001	0.641	<0.001		<0.001	<0.001
	6m vs 24m	Non-self	<0.001	<0.001	<0.001	0.854	0.240	0.171	0.637	<0.001	0.006	<0.001		0.252
	6m vs 24m	Self	0.028	0.088	0.005	0.293	0.707	0.033	0.204	<0.001	0.434	<0.001	0.252	
	12m vs 12m	Non-self	<0.001	<0.001	0.798	<0.001	0.001	<0.001	<0.001	0.955	<0.001	0.660	<0.001	<0.001
	12m vs 24m	Non-self	<0.001	<0.001	0.517	<0.001	<0.001	<0.001	<0.001	0.367	<0.001	0.958	<0.001	<0.001
	12m vs 24m	Self	<0.001	<0.001	0.004	<0.001	<0.001	<0.001	<0.001	<0.001	<0.001	0.007	<0.001	<0.001
	24m vs 24m	Non-self	<0.001	<0.001	0.001	<0.001	<0.001	<0.001	<0.001	<0.001	<0.001	0.002	<0.001	<0.001
Mother	W26-28 vs W26-28	Non-self	<0.001	<0.001	<0.001	<0.001	<0.001	<0.001	<0.001	<0.001	<0.001	<0.001	<0.001	<0.001
	W26-28 vs 6m	Non-self	<0.001	<0.001	<0.001	<0.001	<0.001	<0.001	<0.001	<0.001	<0.001	<0.001	<0.001	<0.001
	W26-28 vs 6m	Self	<0.001	<0.001	<0.001	<0.001	<0.001	<0.001	<0.001	<0.001	<0.001	<0.001	<0.001	<0.001
	6m vs 6m	Non-self	<0.001	<0.001	<0.001	<0.001	<0.001	<0.001	<0.001	<0.001	<0.001	<0.001	<0.001	<0.001

			Child				Mother			
			12m vs 12m	12m vs 24m	12m vs 24m	24m vs 24m	W26-28 vs W26-28	W26-28 vs 6m	W26-28 vs 6m	6m vs 6m
			Non-self	Non-self	Self	Non-self	Non-self	Non-self	Self	Non-self
Child	Neo vs Neo	Non-self	<0.001	<0.001	<0.001	<0.001	<0.001	<0.001	<0.001	<0.001
	Neo vs 6m	Non-self	<0.001	<0.001	<0.001	<0.001	<0.001	<0.001	<0.001	<0.001
	Neo vs 6m	Self	0.798	0.517	0.004	0.001	<0.001	<0.001	<0.001	<0.001
	Neo vs 12m	Non-self	<0.001	<0.001	<0.001	<0.001	<0.001	<0.001	<0.001	<0.001
	Neo vs 12m	Self	0.001	<0.001	<0.001	<0.001	<0.001	<0.001	<0.001	<0.001
	Neo vs 24m	Non-self	<0.001	<0.001	<0.001	<0.001	<0.001	<0.001	<0.001	<0.001
	Neo vs 24m	Self	<0.001	<0.001	<0.001	<0.001	<0.001	<0.001	<0.001	<0.001
	6m vs 6m	Non-self	0.955	0.367	<0.001	<0.001	<0.001	<0.001	<0.001	<0.001
	6m vs 12m	Non-self	<0.001	<0.001	<0.001	<0.001	<0.001	<0.001	<0.001	<0.001
	6m vs 12m	Self	0.660	0.958	0.007	0.002	<0.001	<0.001	<0.001	<0.001
	6m vs 24m	Non-self	<0.001	<0.001	<0.001	<0.001	<0.001	<0.001	<0.001	<0.001
	6m vs 24m	Self	<0.001	<0.001	<0.001	<0.001	<0.001	<0.001	<0.001	<0.001
	12m vs 12m	Non-self		0.391	<0.001	<0.001	<0.001	<0.001	<0.001	<0.001
	12m vs 24m	Non-self	0.391		0.002	<0.001	<0.001	<0.001	<0.001	<0.001
	12m vs 24m	Self	<0.001	0.002		0.516	0.907	0.808	<0.001	0.860
	24m vs 24m	Non-self	<0.001	<0.001	0.516		0.232	0.355	<0.001	0.274
Mother	W26-28 vs W26-28	Non-self	<0.001	<0.001	0.907	0.232		0.776	<0.001	0.893
	W26-28 vs 6m	Non-self	<0.001	<0.001	0.808	0.355	0.776		<0.001	0.876
	W26-28 vs 6m	Self	<0.001	<0.001	<0.001	<0.001	<0.001	<0.001		<0.001
	6m vs 6m	Non-self	<0.001	<0.001	0.860	0.274	0.893	0.876	<0.001	

Supplementary Table S6. P (t-test) from comparisons of the Bray-Curtis distances depicted in Figure 3b. W = gestational week and m = month.

		Gut: child vs mother				Gut: child vs unrelated mother			
		Neonatal	6m	12m	24m	Neonatal	6m	12m	24m
Gut: child vs mother	Neonatal		0.717	<0.001	<0.001	0.436	0.835	<0.001	<0.001
	6m	0.717		<0.001	<0.001	0.212	0.825	<0.001	<0.001
	12m	<0.001	<0.001		<0.001	<0.001	<0.001	0.663	<0.001
	24m	<0.001	<0.001	<0.001		<0.001	<0.001	<0.001	0.137
Gut: child vs unrelated mother	Neonatal	0.436	0.212	<0.001	<0.001		0.179	<0.001	<0.001
	6m	0.835	0.825	<0.001	<0.001	0.179		<0.001	<0.001
	12m	<0.001	<0.001	0.663	<0.001	<0.001	<0.001		<0.001
	24m	<0.001	<0.001	<0.001	0.137	<0.001	<0.001	<0.001	
Oral: child vs mother	6m	<0.001	<0.001	<0.001	<0.001	<0.001	<0.001	<0.001	<0.001
	12m	<0.001	<0.001	<0.001	<0.001	<0.001	<0.001	<0.001	<0.001
	24m	<0.001	<0.001	<0.001	<0.001	<0.001	<0.001	<0.001	<0.001
Oral: child vs unrelated mother	6m	<0.001	<0.001	<0.001	<0.001	<0.001	<0.001	<0.001	<0.001
	12m	<0.001	<0.001	<0.001	<0.001	<0.001	<0.001	<0.001	<0.001
	24m	<0.001	<0.001	<0.001	<0.001	<0.001	<0.001	<0.001	<0.001
Child: gut vs oral (self)	6m	0.492	0.254	<0.001	<0.001	0.918	0.235	<0.001	<0.001
	12m	0.003	0.001	<0.001	<0.001	0.001	<0.001	<0.001	<0.001
	24m	<0.001	<0.001	<0.001	<0.001	<0.001	<0.001	<0.001	<0.001
Child: gut vs oral (non-self)	6m	0.460	0.226	<0.001	<0.001	0.943	0.193	<0.001	<0.001
	12m	0.002	<0.001	<0.001	<0.001	<0.001	<0.001	<0.001	<0.001
	24m	<0.001	<0.001	<0.001	<0.001	<0.001	<0.001	<0.001	<0.001
Mother: gut vs oral (self)	W26-28	<0.001	<0.001	<0.001	<0.001	<0.001	<0.001	<0.001	<0.001
Mother: gut vs oral (non-self)	W26-28	<0.001	<0.001	<0.001	<0.001	<0.001	<0.001	<0.001	<0.001

		Oral: child vs mother			Oral: child vs unrelated mother		
		6m	12m	24m	6m	12m	24m
Gut: child vs mother	Neonatal	<0.001	<0.001	<0.001	<0.001	<0.001	<0.001
	6m	<0.001	<0.001	<0.001	<0.001	<0.001	<0.001
	12m	<0.001	<0.001	<0.001	<0.001	<0.001	<0.001
	24m	<0.001	<0.001	<0.001	<0.001	<0.001	<0.001
Gut: child vs unrelated mother	Neonatal	<0.001	<0.001	<0.001	<0.001	<0.001	<0.001
	6m	<0.001	<0.001	<0.001	<0.001	<0.001	<0.001
	12m	<0.001	<0.001	<0.001	<0.001	<0.001	<0.001
	24m	<0.001	<0.001	<0.001	<0.001	<0.001	<0.001
Oral: child vs mother	6m		0.009	0.087	0.719	0.136	0.361
	12m	0.009		0.411	<0.001	0.056	0.011
	24m	0.087	0.411		0.011	0.510	0.213
Oral: child vs unrelated mother	6m	0.719	<0.001	0.011		0.003	0.048
	12m	0.136	0.056	0.510	0.003		0.357
	24m	0.361	0.011	0.213	0.048	0.357	
Child: gut vs oral (self)	6m	<0.001	<0.001	<0.001	<0.001	<0.001	<0.001
	12m	<0.001	<0.001	<0.001	<0.001	<0.001	<0.001
	24m	<0.001	<0.001	<0.001	<0.001	<0.001	<0.001
Child: gut vs oral (non-self)	6m	<0.001	<0.001	<0.001	<0.001	<0.001	<0.001
	12m	<0.001	<0.001	<0.001	<0.001	<0.001	<0.001
	24m	<0.001	<0.001	<0.001	<0.001	<0.001	<0.001
Mother: gut vs oral (self)	W26-28	<0.001	<0.001	<0.001	<0.001	<0.001	<0.001
Mother: gut vs oral (non-self)	W26-28	<0.001	<0.001	<0.001	<0.001	<0.001	<0.001

		Child: gut vs oral (self)			Child: gut vs oral (non-self)			Mother: gut vs oral (self)	Mother: gut vs oral (non-self)
		6m	12m	24m	6m	12m	24m	W26-28	W26-28
Gut: child vs mother	Neonatal	0.492	0.003	<0.001	0.460	0.002	<0.001	<0.001	<0.001
	6m	0.254	0.001	<0.001	0.226	<0.001	<0.001	<0.001	<0.001
	12m	<0.001	<0.001	<0.001	<0.001	<0.001	<0.001	<0.001	<0.001
	24m	<0.001	<0.001	<0.001	<0.001	<0.001	<0.001	<0.001	<0.001
Gut: child vs unrelated mother	Neonatal	0.918	0.001	<0.001	0.943	<0.001	<0.001	<0.001	<0.001
	6m	0.235	<0.001	<0.001	0.193	<0.001	<0.001	<0.001	<0.001
	12m	<0.001	<0.001	<0.001	<0.001	<0.001	<0.001	<0.001	<0.001
	24m	<0.001	<0.001	<0.001	<0.001	<0.001	<0.001	<0.001	<0.001
Oral: child vs mother	6m	<0.001	<0.001	<0.001	<0.001	<0.001	<0.001	<0.001	<0.001
	12m	<0.001	<0.001	<0.001	<0.001	<0.001	<0.001	<0.001	<0.001
	24m	<0.001	<0.001	<0.001	<0.001	<0.001	<0.001	<0.001	<0.001
Oral: child vs unrelated mother	6m	<0.001	<0.001	<0.001	<0.001	<0.001	<0.001	<0.001	<0.001
	12m	<0.001	<0.001	<0.001	<0.001	<0.001	<0.001	<0.001	<0.001
	24m	<0.001	<0.001	<0.001	<0.001	<0.001	<0.001	<0.001	<0.001
Child: gut vs oral (self)	6m		0.001	<0.001	0.971	<0.001	<0.001	<0.001	<0.001
	12m	0.001		0.013	<0.001	0.947	0.020	0.044	0.033
	24m	<0.001	0.013		<0.001	<0.001	0.617	0.368	0.326
Child: gut vs oral (non-self)	6m	0.971	<0.001	<0.001		<0.001	<0.001	<0.001	<0.001
	12m	<0.001	0.947	<0.001	<0.001		<0.001	0.002	0.001
	24m	<0.001	0.020	0.617	<0.001	<0.001		0.595	0.607
Mother: gut vs oral (self)	W26-28	<0.001	0.044	0.368	<0.001	0.002	0.595		0.905
Mother: gut vs oral (non-self)	W26-28	<0.001	0.033	0.326	<0.001	0.001	0.607	0.905	

Supplementary Table S7. P (t-test) from comparisons of the Jaccard distances presented in Supplementary Table 3. W = gestational week, m = month and neo=neonatal.

			Child											
			Neo vs Neo	Neo vs 6m	Neo vs 6m	Neo vs 12m	Neo vs 12m	Neo vs 24m	Neo vs 24m	6m vs 6m	6m vs 12m	6m vs 12m	6m vs 24m	6m vs 24m
			Non-self	Non-self	Self	Non-self	Self	Non-self	Self	Non-self	Non-self	Self	Non-self	Self
Child	Neo vs Neo	Non-self		<0.001	0.024	<0.001	0.004	<0.001	<0.001	<0.001	0.659	<0.001	<0.001	0.005
	Neo vs 6m	Non-self	<0.001		<0.001	<0.001	0.306	<0.001	<0.001	<0.001	0.007	<0.001	0.004	0.476
	Neo vs 6m	Self	0.024	<0.001		<0.001	<0.001	<0.001	<0.001	0.141	0.017	0.200	<0.001	<0.001
	Neo vs 12m	Non-self	<0.001	<0.001	<0.001		0.164	0.054	0.098	<0.001	<0.001	<0.001	0.565	0.050
	Neo vs 12m	Self	0.004	0.306	<0.001	0.164		0.012	0.019	<0.001	0.011	<0.001	0.352	0.752
	Neo vs 24m	Non-self	<0.001	<0.001	<0.001	0.054	0.012		0.980	<0.001	<0.001	<0.001	0.029	0.002
	Neo vs 24m	Self	<0.001	<0.001	<0.001	0.098	0.019	0.980		<0.001	<0.001	<0.001	0.052	0.003
	6m vs 6m	Non-self	<0.001	<0.001	0.141	<0.001	<0.001	<0.001	<0.001		<0.001	0.850	<0.001	<0.001
	6m vs 12m	Non-self	0.659	0.007	0.017	<0.001	0.011	<0.001	<0.001	<0.001		<0.001	<0.001	0.016
	6m vs 12m	Self	<0.001	<0.001	0.200	<0.001	<0.001	<0.001	<0.001	0.850	<0.001		<0.001	<0.001
	6m vs 24m	Non-self	<0.001	0.004	<0.001	0.565	0.352	0.029	0.052	<0.001	<0.001	<0.001		0.159
	6m vs 24m	Self	0.005	0.476	<0.001	0.050	0.752	0.002	0.003	<0.001	0.016	<0.001	0.159	
	12m vs 12m	Non-self	<0.001	<0.001	0.020	<0.001	<0.001	<0.001	<0.001	0.074	<0.001	0.528	<0.001	<0.001
	12m vs 24m	Non-self	<0.001	<0.001	0.063	<0.001	<0.001	<0.001	<0.001	0.428	<0.001	0.755	<0.001	<0.001
	12m vs 24m	Self	<0.001	<0.001	<0.001	<0.001	<0.001	<0.001	<0.001	<0.001	<0.001	0.001	<0.001	<0.001
	24m vs 24m	Non-self	<0.001	<0.001	<0.001	<0.001	<0.001	<0.001	<0.001	<0.001	<0.001	<0.001	<0.001	<0.001
Mother	W26-28 vs W26-28	Non-self	<0.001	<0.001	<0.001	<0.001	<0.001	<0.001	<0.001	<0.001	<0.001	<0.001	<0.001	<0.001
	W26-28 vs 6m	Non-self	<0.001	<0.001	<0.001	<0.001	<0.001	<0.001	<0.001	<0.001	<0.001	<0.001	<0.001	<0.001
	W26-28 vs 6m	Self	<0.001	<0.001	<0.001	<0.001	<0.001	<0.001	<0.001	<0.001	<0.001	<0.001	<0.001	<0.001
	6m vs 6m	Non-self	<0.001	<0.001	<0.001	<0.001	<0.001	<0.001	<0.001	<0.001	<0.001	<0.001	<0.001	<0.001

			Child				Mother			
			12m vs 12m	12m vs 24m	12m vs 24m	24m vs 24m	W26-28 vs W26-28	W26-28 vs 6m	W26-28 vs 6m	6m vs 6m
			Non-self	Non-self	Self	Non-self	Non-self	Non-self	Self	Non-self
Child	Neo vs Neo	Non-self	<0.001	<0.001	<0.001	<0.001	<0.001	<0.001	<0.001	<0.001
	Neo vs 6m	Non-self	<0.001	<0.001	<0.001	<0.001	<0.001	<0.001	<0.001	<0.001
	Neo vs 6m	Self	0.020	0.063	<0.001	<0.001	<0.001	<0.001	<0.001	<0.001
	Neo vs 12m	Non-self	<0.001	<0.001	<0.001	<0.001	<0.001	<0.001	<0.001	<0.001
	Neo vs 12m	Self	<0.001	<0.001	<0.001	<0.001	<0.001	<0.001	<0.001	<0.001
	Neo vs 24m	Non-self	<0.001	<0.001	<0.001	<0.001	<0.001	<0.001	<0.001	<0.001
	Neo vs 24m	Self	<0.001	<0.001	<0.001	<0.001	<0.001	<0.001	<0.001	<0.001
	6m vs 6m	Non-self	0.074	0.428	<0.001	<0.001	<0.001	<0.001	<0.001	<0.001
	6m vs 12m	Non-self	<0.001	<0.001	<0.001	<0.001	<0.001	<0.001	<0.001	<0.001
	6m vs 12m	Self	0.528	0.755	0.001	<0.001	<0.001	<0.001	<0.001	<0.001
	6m vs 24m	Non-self	<0.001	<0.001	<0.001	<0.001	<0.001	<0.001	<0.001	<0.001
	6m vs 24m	Self	<0.001	<0.001	<0.001	<0.001	<0.001	<0.001	<0.001	<0.001
	12m vs 12m	Non-self		0.640	<0.001	<0.001	<0.001	<0.001	<0.001	<0.001
	12m vs 24m	Non-self	0.640		<0.001	<0.001	<0.001	<0.001	<0.001	<0.001
	12m vs 24m	Self	<0.001	<0.001		0.793	0.068	0.148	<0.001	0.219
	24m vs 24m	Non-self	<0.001	<0.001	0.793		<0.001	0.006	<0.001	0.028
Mother	W26-28 vs W26-28	Non-self	<0.001	<0.001	0.068	<0.001		0.374	<0.001	0.178
	W26-28 vs 6m	Non-self	<0.001	<0.001	0.148	0.006	0.374		<0.001	0.626
	W26-28 vs 6m	Self	<0.001	<0.001	<0.001	<0.001	<0.001	<0.001		<0.001
	6m vs 6m	Non-self	<0.001	<0.001	0.219	0.028	0.178	0.626	<0.001	

Supplementary Table S8. P (t-test) from comparisons of the Jaccard distances presented in Supplementary table 4. W = gestational week and m = month.

		Gut: child vs mother				Gut: child vs unrelated mother			
		Neonatal	6m	12m	24m	Neonatal	6m	12m	24m
Gut: child vs mother	Neonatal		0.451	<0.001	<0.001	0.743	0.366	<0.001	<0.001
	6m	0.451		<0.001	<0.001	0.249	0.894	<0.001	<0.001
	12m	<0.001	<0.001		<0.001	<0.001	<0.001	0.857	<0.001
	24m	<0.001	<0.001	<0.001		<0.001	<0.001	<0.001	0.117
Gut: child vs unrelated mother	Neonatal	0.743	0.249	<0.001	<0.001		0.183	<0.001	<0.001
	6m	0.366	0.894	<0.001	<0.001	0.183		<0.001	<0.001
	12m	<0.001	<0.001	0.857	<0.001	<0.001	<0.001		<0.001
	24m	<0.001	<0.001	<0.001	0.117	<0.001	<0.001	<0.001	
Oral: child vs mother	6m	<0.001	<0.001	<0.001	0.403	<0.001	<0.001	<0.001	0.047
	12m	<0.001	<0.001	<0.001	<0.001	<0.001	<0.001	<0.001	<0.001
	24m	<0.001	<0.001	<0.001	<0.001	<0.001	<0.001	<0.001	<0.001
Oral: child vs unrelated mother	6m	<0.001	<0.001	<0.001	0.568	<0.001	<0.001	<0.001	0.042
	12m	<0.001	<0.001	<0.001	<0.001	<0.001	<0.001	<0.001	<0.001
	24m	<0.001	<0.001	<0.001	<0.001	<0.001	<0.001	<0.001	<0.001
Child: gut vs oral (self)	6m	0.760	0.628	<0.001	<0.001	0.493	0.523	<0.001	<0.001
	12m	<0.001	<0.001	<0.001	<0.001	<0.001	<0.001	<0.001	<0.001
	24m	<0.001	<0.001	<0.001	<0.001	<0.001	<0.001	<0.001	<0.001
Child: gut vs oral (non-self)	6m	0.390	0.073	<0.001	<0.001	0.574	0.043	<0.001	<0.001
	12m	<0.001	<0.001	<0.001	<0.001	<0.001	<0.001	<0.001	<0.001
	24m	<0.001	<0.001	<0.001	<0.001	<0.001	<0.001	<0.001	<0.001
Mother: gut vs oral (self)	W26-28	<0.001	<0.001	<0.001	<0.001	<0.001	<0.001	<0.001	<0.001
Mother: gut vs oral (non-self)	W26-28	<0.001	<0.001	<0.001	<0.001	<0.001	<0.001	<0.001	<0.001

		Oral: child vs mother			Oral: child vs unrelated mother		
		6m	12m	24m	6m	12m	24m
Gut: child vs mother	Neonatal	<0.001	<0.001	<0.001	<0.001	<0.001	<0.001
	6m	<0.001	<0.001	<0.001	<0.001	<0.001	<0.001
	12m	<0.001	<0.001	<0.001	<0.001	<0.001	<0.001
	24m	0.403	<0.001	<0.001	0.568	<0.001	<0.001
Gut: child vs unrelated mother	Neonatal	<0.001	<0.001	<0.001	<0.001	<0.001	<0.001
	6m	<0.001	<0.001	<0.001	<0.001	<0.001	<0.001
	12m	<0.001	<0.001	<0.001	<0.001	<0.001	<0.001
	24m	0.047	<0.001	<0.001	0.042	<0.001	<0.001
Oral: child vs mother	6m		0.003	<0.001	0.732	0.009	<0.001
	12m	0.003		0.321	<0.001	0.507	0.533
	24m	<0.001	0.321		<0.001	0.085	0.628
Oral: child vs unrelated mother	6m	0.732	<0.001	<0.001		0.001	<0.001
	12m	0.009	0.507	0.085	0.001		0.151
	24m	<0.001	0.533	0.628	<0.001	0.151	
Child: gut vs oral (self)	6m	<0.001	<0.001	<0.001	<0.001	<0.001	<0.001
	12m	<0.001	<0.001	<0.001	<0.001	<0.001	<0.001
	24m	<0.001	<0.001	<0.001	<0.001	<0.001	<0.001
Child: gut vs oral (non-self)	6m	<0.001	<0.001	<0.001	<0.001	<0.001	<0.001
	12m	<0.001	<0.001	<0.001	<0.001	<0.001	<0.001
	24m	<0.001	<0.001	<0.001	<0.001	<0.001	<0.001
Mother: gut vs oral (self)	W26-28	<0.001	<0.001	<0.001	<0.001	<0.001	<0.001
Mother: gut vs oral (non-self)	W26-28	<0.001	<0.001	<0.001	<0.001	<0.001	<0.001

		Child: gut vs oral (self)			Child: gut vs oral (non-self)			Mother: gut vs oral (self)	Mother: gut vs oral (non-self)
		6m	12m	24m	6m	12m	24m	W26-28	W26-28
Gut: child vs mother	Neonatal	0.760	<0.001	<0.001	0.390	<0.001	<0.001	<0.001	<0.001
	6m	0.628	<0.001	<0.001	0.073	<0.001	<0.001	<0.001	<0.001
	12m	<0.001	<0.001	<0.001	<0.001	<0.001	<0.001	<0.001	<0.001
	24m	<0.001	<0.001	<0.001	<0.001	<0.001	<0.001	<0.001	<0.001
Gut: child vs unrelated mother	Neonatal	0.493	<0.001	<0.001	0.574	<0.001	<0.001	<0.001	<0.001
	6m	0.523	<0.001	<0.001	0.043	<0.001	<0.001	<0.001	<0.001
	12m	<0.001	<0.001	<0.001	<0.001	<0.001	<0.001	<0.001	<0.001
	24m	<0.001	<0.001	<0.001	<0.001	<0.001	<0.001	<0.001	<0.001
Oral: child vs mother	6m	<0.001	<0.001	<0.001	<0.001	<0.001	<0.001	<0.001	<0.001
	12m	<0.001	<0.001	<0.001	<0.001	<0.001	<0.001	<0.001	<0.001
	24m	<0.001	<0.001	<0.001	<0.001	<0.001	<0.001	<0.001	<0.001
Oral: child vs unrelated mother	6m	<0.001	<0.001	<0.001	<0.001	<0.001	<0.001	<0.001	<0.001
	12m	<0.001	<0.001	<0.001	<0.001	<0.001	<0.001	<0.001	<0.001
	24m	<0.001	<0.001	<0.001	<0.001	<0.001	<0.001	<0.001	<0.001
Child: gut vs oral (self)	6m		<0.001	<0.001	0.190	<0.001	<0.001	<0.001	<0.001
	12m	<0.001		0.024	<0.001	0.639	0.008	0.001	<0.001
	24m	<0.001	0.024		<0.001	0.022	0.829	0.218	0.092
Child: gut vs oral (non-self)	6m	0.190	<0.001	<0.001		<0.001	<0.001	<0.001	<0.001
	12m	<0.001	0.639	0.022	<0.001		0.001	<0.001	<0.001
	24m	<0.001	0.008	0.829	<0.001	0.001		0.064	<0.001
Mother: gut vs oral (self)	W26-28	<0.001	0.001	0.218	<0.001	<0.001	0.064		0.910
Mother: gut vs oral (non-self)	W26-28	<0.001	<0.001	0.092	<0.001	<0.001	<0.001	0.910	

Supplementary Table S9. Shared operational taxonomic units (OTUs) between faecal and saliva samples in children. The number of children, at each sampling occasion, with a specific OTU detected in both the faecal and saliva sample. M = month.

**Taxonomy**	**OTU number**	**6 m n=50**	**12 m n=44**	**24 m n=35**
Firmicutes;Negativicutes;Selenomonadales;Veillonellaceae;Veillonella	5	46	36	15
Firmicutes;Bacilli;Lactobacillales;Streptococcaceae;Streptococcus	3	37	18	16
Firmicutes;Bacilli;Lactobacillales;Streptococcaceae;Streptococcus	17	35	31	28
Firmicutes;Negativicutes;Selenomonadales;Veillonellaceae;Veillonella	183	34	15	2
Proteobacteria;Gammaproteobacteria;Pasteurellales;Pasteurellaceae;Haemophilus	6	31	35	28
Firmicutes;Negativicutes;Selenomonadales;Veillonellaceae;Veillonella	30	28	15	1
Firmicutes;Negativicutes;Selenomonadales;Veillonellaceae;Veillonella	256	20	9	.
Actinobacteria;Actinobacteria;Micrococcales;Micrococcaceae;Rothia	16	18	4	1
Firmicutes;Bacilli;Lactobacillales;Streptococcaceae;Streptococcus	53	18	8	7
Firmicutes;Negativicutes;Selenomonadales;Veillonellaceae;Veillonella	420	17	7	.
Firmicutes;Bacilli;Lactobacillales;Lactobacillales_unclassified;Lactobacillales_unclassified	567	14	6	.
Firmicutes;Negativicutes;Selenomonadales;Veillonellaceae;Veillonella	76	13	11	5
Bacteroidetes;Bacteroidia;Bacteroidales;Bacteroidaceae;Bacteroides	1	12	10	8
Firmicutes;Bacilli;Lactobacillales;Lactobacillales_unclassified;Lactobacillales_unclassified	166	12	2	.
Firmicutes;Clostridia;Clostridiales;Ruminococcaceae;Faecalibacterium	8	10	11	13
Actinobacteria;Actinobacteria;Actinomycetales;Actinomycetaceae;Actinomyces	222	9	2	2
Firmicutes;Bacilli;Lactobacillales;Carnobacteriaceae;Carnobacteriaceae_unclassified	613	9	1	.
Firmicutes;Bacilli;Lactobacillales;Streptococcaceae;Streptococcus	469	9	6	2
Firmicutes;Negativicutes;Selenomonadales;Veillonellaceae;Megasphaera	235	9	6	.
Proteobacteria;Gammaproteobacteria;Betaproteobacteriales;Neisseriaceae;Neisseria	9	9	3	3
Proteobacteria;Gammaproteobacteria;Enterobacteriales;Enterobacteriaceae;Escherichia-Shigella	2	9	8	1
Firmicutes;Bacilli;Lactobacillales;Streptococcaceae;Streptococcus	163	8	4	1
Firmicutes;Clostridia;Clostridiales;Lachnospiraceae;Agathobacter	13	8	9	13
Fusobacteria;Fusobacteriia;Fusobacteriales;Fusobacteriaceae;Fusobacterium	24	8	8	1
Epsilonbacteraeota;Campylobacteria;Campylobacterales;Campylobacteraceae;Campylobacter	70	7	1	1

Taxonomy	OTU number	6 m n=50	12 m n=44	24 m n=35
Firmicutes;Bacilli;Lactobacillales;Streptococcaceae;Streptococcus	155	7	3	.
Firmicutes;Negativicutes;Selenomonadales;Veillonellaceae;Veillonella	791	7	3	.
Proteobacteria;Gammaproteobacteria;Pasteurellales;Pasteurellaceae;Haemophilus	115	7	2	1
Actinobacteria;Actinobacteria;Bifidobacteriales;Bifidobacteriaceae;Bifidobacterium	7	6	2	7
Firmicutes;Bacilli;Lactobacillales;Carnobacteriaceae;Granulicatella	75	6	7	3
Firmicutes;Negativicutes;Selenomonadales;Veillonellaceae;Veillonella	464	6	1	.
Actinobacteria;Actinobacteria;Bifidobacteriales;Bifidobacteriaceae;Bifidobacterium	21	5	6	8
Firmicutes;Bacilli;Bacillales;Family_XI;Gemella	74	5	2	2
Firmicutes;Bacilli;Bacillales;Staphylococcaceae;Staphylococcus	20	5	2	.
Firmicutes;Clostridia;Clostridiales;Lachnospiraceae;Anaerostipes	31	5	6	5
Firmicutes;Negativicutes;Selenomonadales;Veillonellaceae;Veillonella	758	5	1	.
Proteobacteria;Gammaproteobacteria;Enterobacteriales;Enterobacteriaceae;Enterobacteriaceae_unclas	19	5	6	.
Proteobacteria;Gammaproteobacteria;Pasteurellales;Pasteurellaceae;Haemophilus	120	5	.	2
Proteobacteria;Gammaproteobacteria;Pasteurellales;Pasteurellaceae;Pasteurellaceae_unclassified	867	5	3	.
Actinobacteria;Actinobacteria;Bifidobacteriales;Bifidobacteriaceae;Bifidobacteriaceae_unclassifie	1019	4	2	.
Bacteroidetes;Bacteroidia;Bacteroidales;Bacteroidaceae;Bacteroides	10	4	3	3
Firmicutes;Bacilli;Lactobacillales;Lactobacillales_unclassified;Lactobacillales_unclassified	371	4	.	1
Firmicutes;Negativicutes;Selenomonadales;Veillonellaceae;Veillonella	399	4	2	.
Firmicutes;Negativicutes;Selenomonadales;Veillonellaceae;Veillonella	769	4	4	.
Firmicutes;Negativicutes;Selenomonadales;Veillonellaceae;Veillonella	823	4	1	.
Firmicutes;Negativicutes;Selenomonadales;Veillonellaceae;Veillonella	922	4	1	.
Firmicutes;Bacilli;Lactobacillales;Streptococcaceae;Streptococcus	151	3	3	2
Patescibacteria;Saccharimonadia;Saccharimonadales;Saccharimonadaceae;Saccharimonadaceae_ge	33	3	2	2
Proteobacteria;Gammaproteobacteria;Pasteurellales;Pasteurellaceae;Pasteurellaceae_unclassified	271	3	2	1
Proteobacteria;Gammaproteobacteria;Pasteurellales;Pasteurellaceae;Pasteurellaceae_unclassified	439	3	2	1
Proteobacteria;Gammaproteobacteria;Pasteurellales;Pasteurellaceae;Pasteurellaceae_unclassified	472	3	8	3

**Taxonomy**	**OTU number**	**6 m n=50**	**12 m n=44**	**24 m n=35**
Proteobacteria;Gammaproteobacteria;Pasteurellales;Pasteurellaceae;Pasteurellaceae_unclassified	579	3	3	1
Bacteroidetes;Bacteroidia;Bacteroidales;Bacteroidaceae;Bacteroides	4	2	2	5
Firmicutes;Clostridia;Clostridiales;Lachnospiraceae;Blautia	22	2	7	8
Firmicutes;Clostridia;Clostridiales;Lachnospiraceae;Oribacterium	90	2	2	1
Firmicutes;Clostridia;Clostridiales;Peptostreptococcaceae;Intestinibacter	15	2	1	2
Proteobacteria;Gammaproteobacteria;Pasteurellales;Pasteurellaceae;Haemophilus	252	2	2	4
Proteobacteria;Gammaproteobacteria;Pasteurellales;Pasteurellaceae;Pasteurellaceae_unclassified	316	2	4	2
Proteobacteria;Gammaproteobacteria;Pasteurellales;Pasteurellaceae;Pasteurellaceae_unclassified	529	2	15	11
Bacteroidetes;Bacteroidia;Bacteroidales;Bacteroidaceae;Bacteroides	26	1	2	5
Firmicutes;Clostridia;Clostridiales;Clostridiaceae_;Clostridium_sensu_stricto_	28	1	1	3
Firmicutes;Clostridia;Clostridiales;Lachnospiraceae;Lachnospiraceae_ge	55	1	3	4
Firmicutes;Clostridia;Clostridiales;Lachnospiraceae;Lachnospiraceae_unclassified	117	1	.	4
Firmicutes;Clostridia;Clostridiales;Ruminococcaceae;Subdoligranulum	36	1	1	4
Proteobacteria;Gammaproteobacteria;Pasteurellales;Pasteurellaceae;Actinobacillus	234	1	4	3
Proteobacteria;Gammaproteobacteria;Pasteurellales;Pasteurellaceae;Haemophilus	241	1	2	7
Verrucomicrobia;Verrucomicrobiae;Verrucomicrobiales;Akkermansiaceae;Akkermansia	12	1	1	5
Firmicutes;Clostridia;Clostridiales;Lachnospiraceae;Blautia	51	.	2	5
Firmicutes;Clostridia;Clostridiales;Lachnospiraceae;Fusicatenibacter	44	.	2	6
Firmicutes;Clostridia;Clostridiales;Lachnospiraceae;Roseburia	79	.	.	5
Firmicutes;Clostridia;Clostridiales;Ruminococcaceae;Ruminococcus_	68	.	2	5
Firmicutes;Clostridia;Clostridiales;Ruminococcaceae;Ruminococcus_	165	.	2	5

Supplementary Table S10. Shared operational taxonomic units (OTUs) between faecal and saliva samples in mothers. The number of mothers with a specific OTU detected in both the faecal and saliva sample. W = gestational week.

**Taxonomy**	**OTU number**	**W26-28 n=67**
Firmicutes;Bacilli;Lactobacillales;Streptococcaceae;Streptococcus	17	49
Proteobacteria;Gammaproteobacteria;Pasteurellales;Pasteurellaceae;Haemophilus	6	27
Firmicutes;Negativicutes;Selenomonadales;Veillonellaceae;Veillonella	5	26
Firmicutes;Clostridia;Clostridiales;Ruminococcaceae;Faecalibacterium	8	19
Firmicutes;Bacilli;Lactobacillales;Streptococcaceae;Streptococcus	3	17
Bacteroidetes;Bacteroidia;Bacteroidales;Bacteroidaceae;Bacteroides	1	16
Firmicutes;Clostridia;Clostridiales;Lachnospiraceae;Agathobacter	13	15
Actinobacteria;Actinobacteria;Bifidobacteriales;Bifidobacteriaceae;Bifidobacterium	21	14
Firmicutes;Negativicutes;Selenomonadales;Veillonellaceae;Dialister	91	14
Firmicutes;Negativicutes;Selenomonadales;Veillonellaceae;Veillonella	30	13
Actinobacteria;Actinobacteria;Micrococcales;Micrococcaceae;Rothia	16	10
Firmicutes;Clostridia;Clostridiales;Lachnospiraceae;Blautia	22	10
Firmicutes;Bacilli;Lactobacillales;Streptococcaceae;Streptococcus	53	10
Firmicutes;Clostridia;Clostridiales;Lachnospiraceae;Anaerostipes	31	9
Proteobacteria;Gammaproteobacteria;Pasteurellales;Pasteurellaceae;Pasteurellaceae_unclassified	529	9
Proteobacteria;Gammaproteobacteria;Enterobacteriales;Enterobacteriaceae;Escherichia-Shigella	2	8
Bacteroidetes;Bacteroidia;Bacteroidales;Bacteroidaceae;Bacteroides	10	8
Firmicutes;Clostridia;Clostridiales;Ruminococcaceae;Subdoligranulum	36	8
Firmicutes;Negativicutes;Selenomonadales;Veillonellaceae;Veillonella	183	7
Actinobacteria;Actinobacteria;Actinomycetales;Actinomycetaceae;Actinomyces	222	7
Firmicutes;Clostridia;Clostridiales;Ruminococcaceae;Subdoligranulum	47	6
Actinobacteria;Actinobacteria;Bifidobacteriales;Bifidobacteriaceae;Bifidobacterium	7	5
Bacteroidetes;Bacteroidia;Bacteroidales;Bacteroidaceae;Bacteroides	26	5
Firmicutes;Clostridia;Clostridiales;Lachnospiraceae;Fusicatenibacter	44	5
Firmicutes;Clostridia;Clostridiales;Lachnospiraceae;Blautia	51	5
Firmicutes;Clostridia;Clostridiales;Lachnospiraceae;Lachnospiraceae_ge	55	5
Firmicutes;Bacilli;Lactobacillales;Streptococcaceae;Streptococcus	151	5

Taxonomy	OTU number	W26-28 n=67
Firmicutes;Clostridia;Clostridiales;Lachnospiraceae;Coprococcus_	299	5
Firmicutes;Bacilli;Lactobacillales;Streptococcaceae;Streptococcus	469	5
Firmicutes;Clostridia;Clostridiales;Lachnospiraceae;Lachnospiraceae_ge	27	4
Firmicutes;Clostridia;Clostridiales;Clostridiaceae_;Clostridium_sensu_stricto_	28	4
Bacteroidetes;Bacteroidia;Bacteroidales;Bacteroidaceae;Bacteroides	29	4
Bacteroidetes;Bacteroidia;Bacteroidales;Bacteroidaceae;Bacteroides	35	4
Firmicutes;Clostridia;Clostridiales;Ruminococcaceae;Ruminococcus_	52	4
Firmicutes;Clostridia;Clostridiales;Lachnospiraceae;Roseburia	79	4
Bacteroidetes;Bacteroidia;Bacteroidales;Rikenellaceae;Alistipes	88	4
Firmicutes;Clostridia;Clostridiales;Peptostreptococcaceae;Romboutsia	96	4
Bacteroidetes;Bacteroidia;Bacteroidales;Bacteroidaceae;Bacteroides	123	4
Firmicutes;Clostridia;Clostridiales;Ruminococcaceae;Faecalibacterium	132	4
Firmicutes;Bacilli;Lactobacillales;Streptococcaceae;Streptococcus	163	4
Firmicutes;Clostridia;Clostridiales;Ruminococcaceae;Ruminococcus_	165	4
Firmicutes;Clostridia;Clostridiales;Lachnospiraceae;Lachnospiraceae_ND_group	208	4
Proteobacteria;Gammaproteobacteria;Pasteurellales;Pasteurellaceae;Pasteurellaceae_unclassified	472	4
Firmicutes;Firmicutes_unclassified;Firmicutes_unclassified;Firmicutes_unclassified;Firmicutes_unc	2021	4
Proteobacteria;Gammaproteobacteria;Betaproteobacteriales;Neisseriaceae;Neisseria	9	3
Verrucomicrobia;Verrucomicrobiae;Verrucomicrobiales;Akkermansiaceae;Akkermansia	12	3
Firmicutes;Clostridia;Clostridiales;Peptostreptococcaceae;Intestinibacter	15	3
Firmicutes;Bacilli;Bacillales;Staphylococcaceae;Staphylococcus	20	3
Fusobacteria;Fusobacteriia;Fusobacteriales;Fusobacteriaceae;Fusobacterium	24	3
Firmicutes;Clostridia;Clostridiales;Ruminococcaceae;Ruminococcaceae_UCG-	34	3
Bacteroidetes;Bacteroidia;Bacteroidales;Bacteroidaceae;Bacteroides	37	3
Bacteroidetes;Bacteroidia;Bacteroidales;Rikenellaceae;Alistipes	42	3
Actinobacteria;Actinobacteria;Bifidobacteriales;Bifidobacteriaceae;Bifidobacterium	46	3
Firmicutes;Clostridia;Clostridiales;Ruminococcaceae;Ruminococcus_	68	3
Firmicutes;Bacilli;Lactobacillales;Carnobacteriaceae;Granulicatella	75	3
Bacteroidetes;Bacteroidia;Bacteroidales;Bacteroidaceae;Bacteroides	78	3
Firmicutes;Clostridia;Clostridiales;Lachnospiraceae;Blautia	81	3

Taxonomy	OTU number	W26-28 n=67
Firmicutes;Clostridia;Clostridiales;Lachnospiraceae;Lachnospiraceae_unclassified	98	3
Firmicutes;Clostridia;Clostridiales;Ruminococcaceae;Ruminococcaceae_ge	106	3
Proteobacteria;Gammaproteobacteria;Pasteurellales;Pasteurellaceae;Haemophilus	252	3
Firmicutes;Clostridia;Clostridiales;Lachnospiraceae;Lachnospiraceae_unclassified	284	3
Proteobacteria;Gammaproteobacteria;Pasteurellales;Pasteurellaceae;Pasteurellaceae_unclassified	867	3
Firmicutes;Bacilli;Lactobacillales;Streptococcaceae;Streptococcus	1210	3
Bacteroidetes;Bacteroidia;Bacteroidales;Bacteroidaceae;Bacteroides	4	2
Bacteroidetes;Bacteroidia;Bacteroidales;Porphyromonadaceae;Porphyromonas	38	2
Bacteroidetes;Bacteroidia;Bacteroidales;Bacteroidaceae;Bacteroides	39	2
Firmicutes;Negativicutes;Selenomonadales;Veillonellaceae;Veillonella	56	2
Bacteroidetes;Bacteroidia;Bacteroidales;Bacteroidaceae;Bacteroides	62	2
Bacteroidetes;Bacteroidia;Bacteroidales;Bacteroidaceae;Bacteroides	66	2
Bacteroidetes;Bacteroidia;Bacteroidales;Bacteroidaceae;Bacteroides	67	2
Epsilonbacteraeota;Campylobacteria;Campylobacterales;Campylobacteraceae;Campylobacter	70	2
Firmicutes;Clostridia;Clostridiales;Lachnospiraceae;Lachnospira	71	2
Proteobacteria;Gammaproteobacteria;Betaproteobacteriales;Neisseriaceae;Neisseria	80	2
Bacteroidetes;Bacteroidia;Bacteroidales;Bacteroidaceae;Bacteroides	92	2
Bacteroidetes;Bacteroidia;Bacteroidales;Bacteroidaceae;Bacteroides	102	2
Firmicutes;Clostridia;Clostridiales;Lachnospiraceae;Lachnospiraceae_unclassified	117	2
Bacteroidetes;Bacteroidia;Bacteroidales;Bacteroidaceae;Bacteroides	134	2
Bacteroidetes;Bacteroidia;Bacteroidales;Bacteroidaceae;Bacteroides	158	2
Firmicutes;Clostridia;Clostridiales;Lachnospiraceae;Coprococcus_	170	2
Firmicutes;Clostridia;Clostridiales;Lachnospiraceae;CAG-	203	2
Bacteroidetes;Bacteroidia;Bacteroidales;Bacteroidaceae;Bacteroides	209	2
Firmicutes;Clostridia;Clostridiales;Lachnospiraceae;Lachnospiraceae_unclassified	304	2
Firmicutes;Clostridia;Clostridiales;Lachnospiraceae;Lachnospiraceae_ge	376	2
Firmicutes;Clostridia;Clostridiales;Ruminococcaceae;Ruminococcaceae_unclassified	519	2
Actinobacteria;Actinobacteria;Corynebacteriales;Corynebacteriaceae;Corynebacterium	694	2
Firmicutes;Clostridia;Clostridiales;Peptostreptococcaceae;Peptostreptococcus	841	2

Taxonomy	OTU number	W26-28 n=67
Firmicutes;Bacilli;Lactobacillales;Streptococcaceae;Streptococcus	1117	2
Firmicutes;Bacilli;Lactobacillales;Streptococcaceae;Streptococcus	3869	2
